# Comprehensive study of imidazole-based hydrazones: From design, synthesis, and characterization to in vitro and in silico antiproliferative activity analysis

**DOI:** 10.55730/1300-0527.3741

**Published:** 2025-04-15

**Authors:** Ömer DİLEK

**Affiliations:** Central Research Laboratory Application and Research Center, Isparta University of Applied Sciences, Isparta, Turkiye

**Keywords:** Imidazole, hydrazone, cytotoxic activity, molecular docking, molecular dynamic

## Abstract

The aim of this study was to synthesize novel imidazole-cored hydrazones and investigate their cytotoxic activities against human breast and lung cancer cell lines using in vitro and in silico techniques. For this purpose, 5 novel compounds (6 and 7a–7d) were synthesized. The structures of the synthesized compounds were confirmed using spectroscopic methods. The cytotoxic effects were evaluated against human lung (A549) and breast (MCF-7) cancer cell lines using the MTT assay. All synthesized compounds had higher cytotoxic activity against MCF-7 cells (IC_50_ < 9.262 μM) than the control drug cisplatin. However, their cytotoxic activity against A549 cells (IC_50_ > 2.605 μM) was lower than that of cisplatin. Except for compound 7d in the A549 cell line, the IC_50_ values for all compounds were below 10 μM. Absorption, distribution, metabolism excretion, and toxicity (ADMET) properties showed that all compounds follow Lipinski’s rule of five except for compounds 7b and 7d because of high molecular weight. The binding properties of the synthesized compounds to cancer-related proteins (PDB IDs: 1M17, 2XIR, 1E8X, and 1MP8) were also investigated using the molecular docking technique. All compounds showed higher binding affinity to these proteins than the standards used for comparison (erlotinib, sorafenib, copanlisib, and ifebemtinib). As the 7c compound had higher cytotoxicity against the MCF-7 cancer cell line than the others and 2XIR-compound complexes had higher docking scores than other proteins, a molecular dynamics (MD) simulation study was performed to support the stability of the 2XIR-7c complex. The MD simulation results showed that the complex was stable during the 100 ns simulation. When all studied parameters were evaluated, compound 7c had the most potential for further drug development.

## 1. Introduction

Cancer is the second most common cause of death worldwide [1] and is responsible for 1 out of every 6 deaths worldwide [2]. It is a disease caused by cells that divide uncontrollably and have the potential to spread to other bodily tissues. Prostate, breast, lung, and colon are the most common types of cancer worldwide [3]. While breast cancer is the most common type in women, lung and prostate cancer are the most common in men [4]. The current treatment methods include surgery, radiotherapy, chemotherapy, hormonal, and targeted drug therapy [2]. Owing to the undesirable side effects of these treatment methods, there is intensive research into the development of new anticancer drug candidates worldwide [5].

It is important to predict the properties of drug candidates, as it decreases the time and cost to develop the drug. To that end, in silico techniques such as absorption, distribution, metabolism, excretion, and toxicity (ADMET) assessment and molecular docking are particularly useful. The ADMET framework is used to determine the probable physicochemical features of drug candidates from absorption to excretion by simulating in vivo medium. To identify the detrimental effects of chemicals on people, animals, plants, or the environment, it is necessary to determine their toxicity. This is a crucial phase in the creation of new drugs. In silico toxicity determination methods make use of computational techniques to model, simulate, predict, or analyze the toxicity of various substances. The risk of late-stage drug design failures is reduced using these techniques [6]. Protein-ligand complex bonding modes are ascertained by a molecular docking analysis. This gives more information about the binding properties and interactions between ligands and target proteins, and is used to support experimental studies.

Heterocyclic compounds are cyclic structures that contain at least one heteroatom such as nitrogen (N), oxygen (O), or sulfur (S) in their rings other than the carbon atom. These compounds play an important role in both synthetic organic chemistry and medicinal chemistry [7–11]. More than 85% of all biologically active compounds contain heterocycles in their structure [12]. Among the small molecule cancer drugs approved by the FDA, approximately 60% contain nitrogen heterocycles [13]. Imidazole is an aromatic compound that consists of 5 rings, which contain 2 nitrogen atoms in their unit. Imidazole is polar, easily ionizable, amphoteric, and soluble in water and other polar solvents. This enhances the pharmacokinetic and solubility properties of compounds [14]. Therefore, the imidazole ring is a key building block for new drug designs [15–17]. Imidazole compounds have a broad spectrum of pharmacological properties, such as antifungal [18], antitubercular [19], anticancer [20], antibacterial [21], antiviral [22], and antiepileptic [23]. Dacarbazine, bendamustine hydrochloride, fludarabine phosphate, nilotinib, and ponatinib are small molecule anticancer drugs that contain an imidazole ring in their structures. Aldehyde used in the synthesis of compounds contains an imidazole ring and is used as the starting material in the synthesis of nilotinib. The compounds that are derived from this amine have biological activities [24–30]. Therefore, more studies related to this compound should be performed.

Hydrazones are compounds that contain the -N=NH- group in their structures and can be easily obtained by the reaction of aldehydes or ketones with hydrazines. Acyl hydrazones, unlike hydrazones, contain carbonyl groups in their structures and can be easily obtained by the reaction of aldehydes or ketones with hydrazides [31]. Anticancer properties of hydrazone compounds are well known [32–35].

Vascular endothelial growth factor receptor 2 (VEGFR2) is a receptor protein that plays a crucial role in angiogenesis, the process of forming new blood vessels from existing ones [36]. It is mainly found on the surface of endothelial cells, which line the inside of blood vessels [37]. VEGFR2 is activated by binding to vascular endothelial growth factor (VEGF), a signaling protein that stimulates the growth of blood vessels [38]. Activation of VEGFR2 triggers a cascade of signals inside the cell that promote the proliferation and migration of endothelial cells, leading to the formation of new blood vessels [37]. VEGFR2 is a promising target for antiangiogenic therapies with aim of inhibiting the growth of blood vessels in tumors to starve them of nutrients and oxygen [39]. Epidermal growth factor receptor (EGFR) is a receptor protein that is involved in regulating cell growth, proliferation, and survival [40]. It is found on the surface of many cell types, including epithelial cells that line the surfaces of organs and blood vessels. EGFR is activated by binding to epidermal growth factor (EGF), a signaling protein that is produced in response to cell damage or other stimuli [41]. Activation of EGFR triggers a complex network of signaling pathways that regulate cell division, migration, and survival [42]. Abnormal activation of EGFR is common in many types of cancer and is associated with uncontrolled cell growth and tumor progression [43]. EGFR inhibitors are a class of cancer drugs that target EGFR and are used to treat various types of cancer, including lung cancer, colorectal cancer, and head and neck cancer [44]. Focal adhesion kinase (FAK) is a protein kinase that plays a key role in cell adhesion, migration, and survival [45]. FAK is involved in signaling pathways that regulate cell proliferation and survival [46]. Abnormal activation of FAK is associated with cancer metastasis—the spread of cancer cells from the primary tumor to other parts of the body. FAK inhibitors are a potential treatment for cancer metastasis, as they can block the activity of FAK and inhibit cancer cell migration and invasion [47]. Phosphoinositide 3-kinase (PI3K) is a family of enzymes that phosphorylate inositol lipids, which play critical roles in cell signaling, growth, and survival [48]. PI3K is activated by various cellular stimuli, including growth factors, hormones, and cytokines [49]. Upon activation, PI3K generates lipid signaling molecules that activate downstream signaling pathways, such as the AKT/mTOR pathway that regulate cell proliferation, survival, and metabolism. Abnormal activation of PI3K signaling is commonly observed in cancer and is associated with uncontrolled cell growth and tumor progression [50]. PI3K inhibitors are a class of cancer drugs that target PI3K and are used to treat various types of cancer, including breast cancer, ovarian cancer, and lymphoma [48]. Given the importance of these 4 proteins in cancer, VEGFR2 [51], EGFR [51], PI3K [52], and FAK [53] were used as targets for molecular docking in the current study.

In this study, 5 novel imidazole-containing hydrazone compounds (6 and 7a–7d) were synthesized and characterized. In vitro, cytotoxic activity properties were investigated against breast (MCF-7) and lung (A549) cell lines. The molecular docking and ADMET properties of synthesized compounds were also assessed.

## 2. Material and method

### 2.1. Physical measurements

The chemicals were supplied by Merck (Darmstadt, Germany) and Sigma-Aldrich (St. Louis, MO, USA). Chemicals were used as received. Aluminum sheets precoated with silica gel SIL G/UV254 from MACHEREY-NAGEL GmbH & Co. (Düren, Germany), were used for reactions. The spots were made visible under UV light (254 nm). The melting point was measured in an open glass capillary tube using the Stuart SMP 30 melting point apparatus (Cole-Parmer, Vernon Hills, IL, USA). The temperature of the device was started at 25 °C and was increased to 385 °C with a ramp of 10 °C/min. The values were not corrected. A JEOL 400YH NMR spectrometer (400 MHz) (Tokyo, Japan) was used to record the ^1^H and ^13^C NMR spectra. Chemical shifts were expressed as parts per million (ppm) concerning the residual protons (DMSO: δ 2.50) and carbon resonance (DMSO-*d_6_*: δ 39.52) of the solvent. The NMR peak multiplicities were as follows: s for singlet, d for doublet, t for triplet, q for quartet, and m for multiplet. A PG T80+ double beam spectrophotometer (Leicestershire, UK) was used to record UV-Vis spectra using 1 × 10^−4^ M solutions of compounds 6 and 7a–7d. The Fourier transform infrared spectroscopy (FTIR) spectrum was recorded using the Shimadzu IRSpirit QATR-S (Kyoto, Japan). Mass analyses were performed using Waters RADIAN ASAP direct mass detector (Milford, MA, USA) with acquisition mode: full scan, ionization mode: ASAP+/ASAP, mass range: 100–1200 m/z, cone voltage: 10 V, gas: N_2_, heater temperature: 600 °C isothermal, corona current: 3 μA, and sampling technique: capillary dip. Both positive and negative modes were used to analyze compounds. Compound 7d was not analyzed on this device because of ionization. Instead, Waters ACUITY UPLC H-class with ACUITY QDa detector was used. A Waters ACUITY UPLC BEH C18 50 mm × 2.1 mm × 1.7 μm column was used with mobile phase A: 0.1% formic acid, mobile phase B: ACN, sample temperature: 15 °C, column temperature: 35 °C, QDa parameters: 600 °C probe temperature, one gain, 2 points/min sampling ratio, 0.8 kV positive capillary, positive polarization, and 100–1200 Da mass range. The analysis time was 10 min. Gradient parameters of the system were as follows: initially, 0.4 mL/min (90% A, 10% B), 0.5 min 0.4 mL/min (90% A, 10% B), 5 min 0.4 mL/min (20% A, 80% B), 7 min 0.4 mL/min (20% A, 80% B), and 10 min 0.4 mL/min (90% A, 10% B). The absorbance values of MTT assays were measured using the BioTek Epoch 2 Elisa plate reader (Agilent, Santa Clara, CA, USA) at 590 nm. GraphPad Prism 5 software was used for calculating the IC_50_ values of the compounds tested.

### 2.2. Synthesis

#### 2.2.1. Synthesis of esters (2a, 2c)

Esters 2b and 2d were commercially available. Since esters 2a and 2c were not available in the laboratory, they were synthesized starting from carboxylic acids.

##### 2.2.1.1. Methyl benzoate (2a)

Benzoic acid (1a, 3.05 g, 25.0 mmol, 1.00 eq), H_2_SO_4_ (5–6 drops), and methanol (50 mL) were added into the 2-necked round bottom flask equipped with a magnetic stirring bar and reflux condenser. The mixture was heated to 70 °C via an oil bath. The conversion was followed by TLC. After 6 h, the starting material was completely consumed. The flask was cooled down to ambient temperature. The mixture was poured into a beaker with 50 mL of 5% aqueous NaHCO_3_. The organic contents were extracted with dichloromethane (3 × 50 mL). The combined organic extracts were dried over Na_2_SO_4_, filtered, and concentrated by rotary vacuum evaporation. Compound 2a (3.06 g, 22.5 mmol) was obtained as a colorless oil at 95% yield.

##### 2.2.1.2. Methyl thiophene-3-carboxylate (2c)

Thiophene-3-carboxylic acid (1c, 3.2 g, 25.0 mmol, 1.00 eq), H_2_SO_4_ (5–6 drops), and methanol (50 mL) was used according to the synthesis procedure of 2a. Compound 2c (3.27 g, 23.0 mmol) was obtained as a colorless oil at 92% yield.

#### 2.2.2. Synthesis of hydrazides (3a–d)

##### 2.2.2.1. Benzhydrazide (3a)

Methyl benzoate (2a, 2.72 g, 20.0 mmol, 1.00 eq) and hydrazine hydrate (85% in water) (45 mL) were added into a 2-necked round bottom flask equipped with a magnetic stirring bar and reflux condenser. The mixture was heated to 120 °C via an oil bath. TLC was used to follow the conversion. After 2 h, the starting material was completely consumed. The flask was cooled down to ambient temperature. The remaining hydrazine hydrate was concentrated by rotary vacuum evaporation. Then, 25 mL of distilled water was added to the mixture. The precipitate was filtered and dried under a vacuum. Compound 3a (2.61 g, 19.2 mmol) was obtained as a white solid at 96% yield. Mp: 114–116 °C.

##### 2.2.2.2. 4-Hydroxybenzhydrazide (3b)

Methyl 4-hydroxybenzoate (2b, 3.04 g, 20.0 mmol, 1.00 eq), and hydrazine hydrate (85% in water) (45 mL) were used according to synthesis procedure of 3a. Compound 3b (2.8 g, 18.4 mmol) was obtained as a white solid at 92% yield. Mp: 263–265 °C.

##### 2.2.2.3. Thiophene-3-carbohydrazide (3c)

Methyl thiophene-3-carboxylate (2c, 2.84 g, 20.0 mmol, 1.00 eq), and hydrazine hydrate (85% in water) (45 mL) were used according to the synthesis procedure of 3a. Compound 3c (2.5 g, 17.6 mmol) was obtained as a white solid at 88% yield. Mp: 122–124 °C.

##### 2.2.2.4. 3,4,5-trihydroxy benzhydrazide (3d)

Ethyl-3,4,5-trihydroxy benzoate (2d, 3.96 g, 20.0 mmol, 1.00 eq), and hydrazine hydrate (85% in water) (45 mL) were used according to the synthesis procedure of 3a. Compound 3d (2.95 g, 16.0 mmol) was obtained as a white solid at 80% yield. Mp: 285–290 °C (decomp).

#### 2.2.3. Synthesis of hydrazones (6 and 7a–7d)

##### 2.2.3.1. 4-((3-(4-Methyl-1H-imidazole-1-yl)-5-(trifluoromethyl)phenyl)diazenyl)-2-((2-phenyl hydrazone)methyl)phenol (6)

A mixture of 2 mmol 2-hydroxy-5-((3-(4-methyl-1*H*-imidazole-1-yl)-5-trifluoromethyl)phenyl)diazenyl)benzaldehyde (4) [51], 2.4 mmol phenylhydrazine (5), and 50 mL absolute ethanol was added to a 100 mL 2-necked flask, along with a magnetic stirring bar. The mixture was stirred and refluxed using a heated magnetic stirrer for at least 4 h under nitrogen and reflux conditions. TLC was used to follow the conversion. After the conversion was completed, half of the solvent was removed under reduced pressure. The flask was put into the refrigerator overnight. The precipitate was filtered and recrystallized with ethanol. A 76% yield (705 mg) of compound 6 was obtained as a brown solid. Mp: 250–252 °C. FTIR (ATR): *ṽ*_max_ (cm^−1^) = 3250 (br, w), 3080 (w), 1600 (s), 1567 (s), 1495 (s), 1303 (s), 1277 (s), 1260 (s), 1172 (s), 1153 (s), 1110 (s), 1072 (s), 1005 (m) (supporting information S20). ^1^H NMR (700 MHz, DMSO): *d* (ppm) 11.33 (s, 1H), 10.59 (s, 1H), 8.45 (s, 1H), 8.37 (s, 1H), 8.30 (d, *J* = 2.4 Hz, 1H), 8.22 (s, 1H), 8.14 (s, 1H), 7.99 (s, 1H), 7.83 (dd, *J* = 8.7, 2.4 Hz, 1H), 7.74 (s, 1H), 7.26 (t, *J* = 7.8 Hz, 2H), 7.10 (d, *J* = 8.7 Hz, 1H), 7.04 (d, *J* = 7.7 Hz, 2H), 6.79 (t, *J* = 7.2 Hz, 1H), 2.19 (s, 3H) (supporting information S3). ^13^C{^1^H} NMR (APT, 176 MHz, DMSO): *d* (ppm) 159.50 (C), 153.56 (C), 145.14 (C), 144.71 (C), 138.99 (C), 138.73 (C), 134.37 (CH), [131.89, 131.70, 131.52, 131.33, (C, *J^2^_C-F_* = 32.7 Hz)], 129.35 (CH), [125.83, 124.28, 122.73, 121.18, (C, *J^1^_C-F_* = 273.6 Hz)], 124.09 (CH), 122.21 (CH), 122.10 (C), 119.23 (CH), 117.86 (CH), [115.53, 117.52, (CH, *J^3^_C-F_* = 2.7 Hz)], 116.86 (CH), [114,95, 114.93, (CH, *J^3^_C-F_* = 3.0 Hz)], 114.31 (CH), 111.98 (2× CH), 13.64 (CH_3_) (supporting information S3). MS: *m/z* = [M+H]^+^ calculated for C_24_H_20_F_3_N_6_O 465.17, found 465.33; *m/z* = [M–H]^−^ calculated for C_24_H_18_F_3_N_6_O 463.15, found 463.13 (supporting information S11–S12).

##### 2.2.3.2. N’-(2-hydroxy-5-((3-(4-methyl-1H-imidazole-1-yl)-5-(trifluoromethyl) phenyl)diazenyl)benzylidene)benzohydrazide (7a)

A mixture of 2 mmol 2-Hydroxy-5-((3-(4-methyl-1*H*-imidazole-1-yl)-5-trifluoromethyl)phenyl)diazenyl)benzaldehyde (4), 2.4 mmol benzyhydrazide (3a), and 50 mL absolute ethanol was used according to the synthesis procedure of 6. An 81% yield (800 mg) of compound 7a was obtained as an orange solid. Mp: 242–244 °C. FTIR (ATR): *ṽ*_max_ (cm^−1^) = 2980 (w), 2800 (w), 1667 (s), 1604 (s), 1577 (m), 1490 (s), 1374 (s), 1323 (s), 1307 (s), 1280 (s), 1113 (s), 1070 (s) (supporting information S20). ^1^H NMR (400 MHz, DMSO-*d_6_*): *d* (ppm) 12.15 (s, 1H, NH), 8.78(s, 1H), 8.48 (s, 1H), 8.40 (s, 1H), 8.33 (s, 1H), 8.14 (s, 1H), 7.97 (d, *J* = 6.7 Hz, 4H), 7.78 (s, 1H), 7.58 (dd, *J* = 21.2, 6.6 Hz, 3H), 7.10 (d, *J* = 8.4 Hz, 1H), 2.18 (s, 3H) (supporting information S4). ^13^C{^1^H} NMR (DEPT, 101 MHz, DMSO-*d_6_*): *d* (ppm) 163.11 (C), 162.71 (C), 153.63 (C), 145.96 (CH), 144.27 (C), 138.96 (C), 138.77 (C), 135.48 (C), 132.93 (C), [132.10, 131.77, 131.44, 131.11, (C, *J^2^_C-F_* = 32.7 Hz)], 132.07 (CH), 128.62 (CH), 127.79 (CH), [127.61, 124.90, 122.18, 119.46, (C, *J^1^_C-F_* = 273.0 Hz)], 27.10 (CH), 123.55 (CH), 120.42 (s), 118.04 (CH), 117.93 (CH), 117.28 (CH), 114.48 (CH), 13.70 (CH_3_) (supporting information S4–S5). MS: *m/z* = [M+H]^+^ calculated for C_25_H_20_F_3_N_6_O_2_ 493.16, found 493.34; *m/z* = [M–H]^−^ calculated for C_25_H_18_F_3_N_6_O_2_ 491.14, found 491.07 (supporting information S13–S14).

##### 2.2.3.3. 4-Hydroxy-N’-(2-hydroxy-5-((3-(4-methyl-1H-imidazole-1-yl)-5-(trifluoro methyl)phenyl)diazenyl)benzylidene)benzohydrazide (7b)

A mixture of 2 mmol 2-Hydroxy-5-((3-(4-methyl-1*H*-imidazole-1-yl)-5-trifluoromethyl)phenyl)diazenyl)benzaldehyde (4), 2.4 mmol 4-Hydroxybenzhydrazide (3b), and 50 mL absolute ethanol was used according to the synthesis procedure of 6. A 70% yield (710 mg) of compound 7b was obtained as an orange solid. Mp: 308–310 °C. FTIR (ATR): *ṽ*_max_ (cm^−1^) = 3567 (w), 3000 (m), 2900 (m), 1651 (s), 1610 (s), 1503 (s), 1280 (s), 1243 (s), 1173 (s), 1098 (s), 1075 (s) (supporting information S21). ^1^H NMR (400 MHz, DMSO-*d_6_*): *d* (ppm) 12.00 (s, 2H), 10.15 (s, 1H), 8.72 (s, 1H), 8.45 (s, 1H), 8.38 (s, 1H), 8.28 (d, *J* = 2.1 Hz, 1H), 8.14 (s, 1H), 7.99 (s, 1H), 7.95 (dd, *J* = 8.8, 2.3 Hz, 1H), 7.86 (s, 1H), 7.84 (s, 1H), 7.74 (s, 1H), 7.15 (d, *J* = 8.8 Hz, 1H), 6.90 (s, 1H), 6.88 (s, 3H), 2.19 (s, 3H)(supporting information S6). ^13^C{^1^H} NMR (DEPT, 101 MHz, DMSO-*d_6_*): *d* (ppm) 162.58 (C=O), 161.08 (C), 160.90 (C), 153.41 (C), 145.26 (CH), 144.79 (C), 138.89 (C), 138.67 (C), [132.03, 131.70, 131.37, 131.04, (C, *J^2^_C-F_* = 33.1 Hz)], 129.74 (CH), [127.44, 124.72, 122.00, 119.28, (C, *J^1^_C-F_* = 273.0 Hz)], 126.06 (CH), 124.11 (CH), 123.14 (C), 119.99 (C), 117.88 (CH), [117.49, 117.52, (CH, *J^3^_C-F_* = 2.9 Hz)], 117.38 (CH), 115.05 (CH), [114.68, 114.65, (CH, *J^3^_C-F_* = 3.2 Hz)], 13.49 (CH_3_) (supporting information S6–7). MS: *m/z* = [M+H]^+^ calculated for C_25_H_20_F_3_N_6_O_3_ 509.16, found 509.34; *m/z* = [M–H]^−^ calculated for C_25_H_18_F_3_N_6_O_3_ 507.14, found 507.14 (supporting information S15–S16).

##### 2.2.3.4. N’-(2-hydroxy-5-((3-(4-methyl-1H-imidazole-1-yl)-5-(trifluoromethyl) phenyl)diazenyl)benzylidene)thiophene-3-carbohydrazide (7c)

A mixture of 2 mmol 2-Hydroxy-5-((3-(4-methyl-1*H*-imidazole-1-yl)-5-trifluoromethyl)phenyl)diazenyl)benzaldehyde (4), 2.4 mmol thiophene-3-carbohydrazide (3c), and 50 mL of absolute ethanol was used according to the synthesis procedure of 6. An 83% yield (830 mg) of compound 7c was obtained as an orange solid. Mp: 243–245 °C. FTIR (ATR): *ṽ*_max_ (cm^−1^) = 3100 (w), 2900 (w), 2800 (w), 1664 (s), 1602 (s), 1484 (s), 1376 (s), 1306 (s), 1279 (s), 1153 (m), 1135 (m), 1113 (s), 1069 (m) (supporting information S21). ^1^H NMR (400 MHz, DMSO-*d_6_*): *d* (ppm) 12.05 (s, 1H), 8.75 (s, 1H), 8.48 (s, 1H), 8.40 (s, 1H), 8.36 (s, 1H), 8.33 (s, 1H), 8.13 (s, 1H), 7.98 (s, 1H), 7.94 (dd, *J* = 8.9, 1.8 Hz, 1H), 7.78 (s, 1H), 7.70–7.66 (m, 1H), 7.64 (d, *J* = 4.7 Hz, 1H), 7.08 (d, *J* = 8.7 Hz, 1H), 2.18 (s, 3H) (supporting information S7). ^13^C{^1^H} NMR (DEPT, 101 MHz, DMSO-*d_6_*): *d* (ppm) 163.36 (s, C=O), 159.12 (C), 154.22 (C), 146.14 (CH), 144.73 (C), 139.28 (C), 136.21 (C), [132.60, 132.28, 131.95, 131.63, (C, *J^2^_C-F_* = 32.7 Hz)], 130.65 (CH), 127.69 (CH), 127.48 (CH), 127.22 (CH), 124.34 (CH), 120.93 (C), [128.08, 125.36, 122.65, 119.93, (C, *J^1^_C-F_* = 272.9 Hz)], 118.47 (CH), 118.35 (CH), [117.76, 117.73 (CH, *J^3^_C-F_* = 3.1 Hz)], 115.17 (CH), 114.88 (CH), 14.13 (CH_3_) (supporting information S8). MS: *m/z* = [M+H]^+^ calculated for C_23_H_18_F_3_N_6_O_2_S 499.12, found 499.27; *m/z* = [M–H]^−^ calculated for C_25_H_16_F_3_N_6_O_3_ 497.10, found 497.07 (supporting information S17–S18).

##### 2.2.3.5. 3,4,5-Trihydroxy-N’-(2-hydroxy-5-((3-(4-methyl-1H-imidazol-1-yl)-5-(trifluoromethyl)phenyl)diazenyl)benzylidene)benzohydrazide (7d)

A mixture of 2 mmol 2-Hydroxy-5-((3-(4-methyl-1*H*-imidazole-1-yl)-5-trifluoromethyl)phenyl)diazenyl)benzaldehyde (4), 2.4 mmol 3,4,5-trihydroxy benzhydrazide (3d), and 50 mL of absolute ethanol was used according to the synthesis procedure of 6. A 74% yield (800 mg) of compound 7d was obtained as a yellow solid. Mp: 252–254 °C. FTIR (ATR): *ṽ*_max_ (cm^−1^) = 3350 (w), 3250 (w), 1654 (m), 1605 (s), 1534 (m), 1491 (m), 1434 (m), 1324 (s), 1274 (s), 1220 (s), 1041(s) (supporting information S22). ^1^H NMR (400 MHz, DMSO-*d_6_*): *d* (ppm) 11.94 (s, 1H), 9.29 (s, 2H), 8.72 (s, 1H), 8.45 (s, 1H), 8.40 (s, 1H), 8.27 (d, *J* = 2.4 Hz, 1H), 8.15 (s, 1H), 8.01 (s, 1H), 7.96 (dd, *J* = 8.8, 2.5 Hz, 1H), 7.85–7.54 (m, 1H), 7.15 (d, *J* = 8.8 Hz, 1H), 6.98 (s, 2H), 6.79 (s, 2H), 2.19 (s, 3H) (supporting information S9). ^13^C{^1^H} NMR (DEPT, 101 MHz, DMSO-*d_6_*): *d* (ppm) 166.37 (C), 163.03 (C), 161.18 (C), 153.47 (C), 145.61 (C), 145.36 (CH), 144.80 (C), 138.71 (C), 137.31 (C), 136.09 (C), 132.07, 131.74, 131.41, 131.08, (C, *J^2^_C-F_* = 33.0 Hz)], [127.45, 124.73, 122.02, 119.30, (C, *J^1^_C-F_* = 272.8 Hz)],126.10 (CH), 124.22 (CH), 123.60 (C), 122.57 (C), 117.91 (CH), [117.58, 117.54, (CH, *J^3^_C-F_* =3.5 Hz)], 117.45 (CH), [114.74, 114.70, (CH, *J^3^_C-F_* = 4.0 Hz)], 107.28 (CH), 106.48 (2× CH), 13.51 (CH_3_), (supporting information S9–10). MS: *m/z* = [M+H]^+^ calculated for C_25_H_20_F_3_N_6_O_5_ 541.47, found 541.05 (supporting information S19).

### 2.3. In vitro cytotoxic activity

Cytotoxic activity of the compounds was assessed according to the procedure described in the literature [54–56]. The human epithelial breast adenocarcinoma cell line (MCF-7, ATCC HTB-22) and human lung cancer cell line (A549, ATCC CCL-185) were purchased from ATCC (Manassas, VA, USA). Cells were cultured in Dulbecco’s modified Eagle’s medium-high-glucose (DMEM) supplemented with 10% fetal bovine serum (FBS) and 1% glutamax. After the cells reached 90% density in the 75 cm^2^ flask, they were lifted from the surface of the flask using 2.5 mL of trypsin EDTA. The cells were transferred to a 15 cm^2^ falcon tube containing 2.5 mL of DMEM and centrifuged. After centrifugation for 5 min at 1450 rpm, the supernatant was discarded and 5 mL of fresh DMEM was placed on the pellet. A 5 mL pipette was used to distribute the cells evenly in DMEM. The cells were counted using a Thoma cell counting chamber. Cultured cells were seeded into a sterile 96-well plate at a density of 4 × 10^3^ cells/well and incubated at 37 °C and 5% CO_2_ with saturating humidity for 24 h. After 24 h, the media was changed and cancer cells were exposed to compounds 6, 7a–7d, and cisplatin at 200, 100, 50, 25, 12.5, and 6.25 μM concentrations for 72 h. The media was removed and MTT stock solution (50 μL, 5 mg/mL) was added into the wells in the sterile 96-well plate and incubated for a further 3 h. Media was removed after 3 h and 200 μL DMSO was added into the wells to dissolve the formed formazan. The sterile 96-well plate was shaken for 15 min to obtain a homogeneous mixture, and the absorbance measurements were performed at 590 nm using an Epoch 2 ELISA plate reader. As in other studies, IC_50_ values were calculated using the GraphPad Prism 5 program [57–59].

### 2.4. Computational analyses

#### 2.4.1. ADMET properties

ADMET calculations of synthesized compounds were investigated using the ADMETlab 2.0 web server [60]. LD_50_ (mg/kg) and toxicity classes of the synthesized compounds were calculated using the Protox-II web server [61].

#### 2.4.2. Geometry optimization

Optimized geometries of the synthesized compounds were calculated using Avogadro software and universal force field (UFF) parameters. [62].

#### 2.4.3. Molecular docking

AutodockVina version 1.1.2 [63] software was used for the molecular docking analysis of the synthesized compounds (6 and 7a–7d) with selected proteins. UCSF Chimera version 1.17.2 [64] software and BIOVIA Discovery Studio Visualizer [65] software were used for 3D and 2D visualizations, respectively. PDB ID:1M17 for EGFR, PDB ID:2XIR for VEGFR2, PDB ID:1E8X for PI3K, and PDB ID:1MP8 for FAK proteins related to cancer research were used in the molecular docking analysis. The crystal structures of the used proteins were taken from the Protein Data Bank as pdb files. UCSF Chimera was used for the removal of water and all nonstandard residues. The Modeller program was used to homology modelling of proteins before molecular docking analyses [66]. A grid box with sizes 45 × 45 × 45 Å^3^ was used to surround the active site of proteins. The binding site coordinates of proteins were calculated using the Deepsite web server [67] and the coordinates are depicted in Table 1. Optimized geometries of synthesized compounds were obtained from Avogadro calculations.

The molecular mechanics Poisson-Boltzmann surface area (MMPBSA) binding energies for all relevant docking analyses were calculated using YASARA software, focusing on protein-ligand complexes with the highest docking scores. Initially, the best docking pose obtained from molecular docking analyses was loaded into YASARA. Molecular dynamics (MD) simulations were initiated using the md_run.mcr macro. Once the first simulation file was generated, the simulation was subsequently halted. Utilizing this initial simulation snapshot, the binding energy was calculated via the md_analyzebindenergy.mcr macro. The binding energy was determined based on the following equation: Binding Energy = E_potRecept_ + E_solvRecept_ + E_potLigand_ + E_solvLigand_ − E_potComplex_ − E_solvComplex_ [68].

#### 2.4.4. Molecular dynamics simulation

MD simulation of the protein-ligand (2XIR-7c) complex was conducted using YASARA [69]. To enhance solute (the protein and the ligand together) stability, the hydrogen bonding network was optimized, and the protonation states of protein residues were adjusted to a pH of 7.4 based on pKa predictions [70,71]. By providing an excess of Na^+^ or Cl^−^ ions to neutralize the cell, a physiological concentration of 0.9% of Na^+^/Cl^−^ ions was created. AMBER14, GAFF2, AM1-BCC, and TIP3P force fields were used for solute, ligand, and water, respectively [72–74]. The simulation ran for 100 ns, incorporating the steepest descent and simulated annealing minimizations to resolve initial conflicts. The van der Waals forces were assigned a cutoff at 8 Å, whereas electrostatic forces were not limited by any cutoff [75,76]. The equations of movements were integrated with a multiple timestep of 2.5 fs for bonded interactions and 5 fs for nonbonded interactions at a temperature of 298 K and a pressure of 1 atm (NPT ensemble) using previously described methodologies [77].

## 3. Results and discussion

### 3.1. Synthesis

One aim of this study was to synthesize hydrazone derivatives. The compounds were well designed, and the retrosynthetic approach was used to synthesize designed products. Hydrazones can be synthesized using aldehyde or ketone and hydrazine, or hydrazides. The azo-containing aldehyde was synthesized in a previous study by our team [51]. This aldehyde derivative was used to synthesize all hydrazone compounds. The literature shows that hydrazide compounds can be synthesized from esters. The esters 2a and 2c were obtained by the reaction of carboxylic acids with methanol in the presence of H_2_SO_4_. Esters 2b and 2d were commercially available. Esters 2a–2d were converted to hydrazide derivatives in good yields (80%–96% yield) under reflux in hydrazine hydrate [78]. The synthesized hydrazides are depicted in Table 2.

The derivative compounds were designed to compare the effect of the carbonyl group, phenolic group, number of phenolic OH, and aromatic group on cytotoxic activity. Compound 6 was synthesized by the reaction of aldehyde and phenyl hydrazine at 76% yield. Benzhydrazide (3a), 4-hydroxybenzydrazide (3b), thiophene-3-carbohydrazide (3c), and 3,4,5-trihydroxybenzhydrazide (3d) compounds were used to synthesize hydrazone 7a–7d derivatives, respectively. The synthesis scheme, substrates, and obtained products are shown in Table 3. All hydrazone compounds were synthesized by reactions with azo-containing aldehyde [51] and suitable amine or hydrazide derivatives in good yields (70%–83%).

### 3.2. UV absorbance measurements

**Figure 1:**
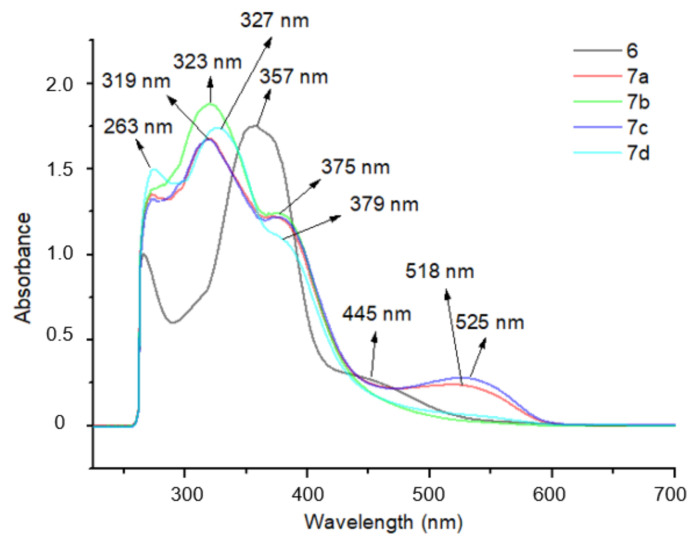
UV-Vis graph of compounds 6 and 7a–7d in DMSO.

UV absorbance measurements of synthesized compounds were performed to determine maximum wavelengths. The 1 × 10^−4^ M solution of synthesized compounds 6 and 7a–7d was prepared in DMSO. These measurements were important as absorbance measurements in the cytotoxic activity assays were conducted at 590 nm in DMSO. There were no maximum wavelengths of 590 nm. The maximum wavelengths are given in Table 4 and UV-Vis spectra are depicted in Figure 1. While compounds 6, 7b, and 7d have 3 maximum absorbances, 7a and 7c have 4. The first maximum absorbance peak of all compounds was 263 nm.

### 3.3. In vitro cytotoxic activity

Chemotherapy is the most common cancer treatment. Due to the side effects of chemotherapeutics, new treatments are still needed. In this study, promising chemotherapeutic compounds were synthesized and investigated for their cytotoxic activities against breast and lung cancer cell lines. Cisplatin was used as a control drug for a meaningful comparison. The results are given in Table 5.

The cytotoxic activity results can be interpreted in the context of the structural differences between the compounds. In the MCF-7 cell line, compound 7a had higher cytotoxic activity (IC_50_ = 0.6402 μM) compared to compound 6 (IC_50_ = 0.6642 μM) due to the addition of a carbonyl group in the structure of 7a. The presence of one phenolic -OH group in compound 7b decreased the cytotoxic activity (IC_50_ = 0.7230 μM) in the MCF-7 cell line. The presence of 3 phenolic -OH groups in compound 7d further decreased cytotoxic activity (IC_50_ = 0.8203 μM). Thus, as the number of phenolic -OH groups increase, the cytotoxic activity appears to decrease in the MCF-7 cell line. Compound 7c had thiophene as an aromatic ring and this compound had the highest cytotoxic activity (IC_50_ = 0.6312 μM) in the MCF-7 cell line. All synthesized compounds had higher cytotoxic activity than the control drug (IC_50_ = 9.262 μM) in the MCF-7 cell line.

Conversely, all synthesized compounds had lower cytotoxic activity than the control drug in the A594 cell line. The addition of the carbonyl group in compound 7a increased the cytotoxic activity compared to compound 6 (IC_50_ = 4.499 μM and IC_50_ = 5.580 μM, respectively). The singular phenolic -OH group increased the cytotoxic activity in compound 7b (IC_50_ = 3.472 μM). This was the highest cytotoxic activity and the closest to the control drug (IC_50_ = 2.605 μM). The addition of more phenolic -OH groups in compound 7d drastically decreased cytotoxic activity (IC_50_ = 29.07 μM). When the aromatic ring was changed to thiophene, the cytotoxic activity was lower (IC_50_ = 4.056 μM). While the cytotoxic activity of all synthesized compounds was lower than the control drug in the A594 cell line, values were lower still than 10 μM with the exception of 7d.

### 3.4. Computational analyses

#### 3.4.1. ADMET properties

Establishing the ADMET parameters is important in medicinal chemistry to assess the suitability of new drug candidates. The ADMET parameters of synthesized compounds were calculated using the ADMETlab 2.0 web server [60]. The results are given in Table 6. Physicochemical properties like molecular weight, number of hydrogen bond acceptors and donors, number of heteroatoms, ring, rotatable bonds, formal charge, rigid bond, topological polar surface area (TPSA), logS (water solubility), logP (octanol-water partition coefficient), and logD (logP at physiological pH 7.4). The molecular weight should be less than 500 Da. Compounds 6, 7a, and 7c met the molecular weight criteria, while 7b and 7d were higher than 500 Da. The TPSA value should be less than 140 Å^2^ [79]. All synthesized compounds were less than 140 Å^2^, except for 7d.

According to Lipinski’s rule of five in medicinal chemistry: “the number of hydrogen bond donors should be greater than 5. The number of hydrogen bond acceptors should not be greater than 10. The molecular weight should not be greater than 500, and logP should not be greater than 5.” If 2 properties are out of range, poor absorption or permeability is a risk. One property out of range is acceptable [80]. While compounds 6, 7a, and 7c meet the criteria for Lipinski’s rule of five, molecules 7b and 7d do not because their molecular weight is too high.

Oral medication needs to cross intestinal cell membranes by passive diffusion, carrier-mediated uptake, or active transport mechanisms before it can enter the systemic circulation. The human colon adenocarcinoma cell line (Caco-2) has been widely used to estimate in vivo drug permeability because of its morphological and functional similarities to human intestinal epithelium. As a result, Caco-2 cell permeability has emerged as a crucial metric for qualifying medicinal candidates. It is recommended that the Caco-2 permeability value be higher than −5.15 [60]. All Caco-2 permeability values of synthesized compounds were higher than −5.15, except for 7d.

The Ames toxicity test is used to predict the carcinogenicity of drug candidates. The Ames toxicity values for all synthesized compounds were within the desired range, except for 7c. Assessing the acute toxicity of potential drugs in mammals, such as rats or mice, is crucial for the safety assessment process. The toxicity class and LD_50_ values were also calculated by using the Protox-II web server. Compound 6 was fourth class in the toxicity classification (LD_50_ = 1000 mg/kg) while the other compounds (7a–7d) were third class (LD_50_ = 125 mg/kg). This means that compound 6 is less toxic than the others.

#### 3.4.2. Molecular docking

Cancer-related proteins with PDB IDs 1M17, 2XIR, 1E8X, and 1MP8 (EGFR, VEGFR2, PI3K, and FAK, respectively) were selected for molecular docking analysis. All synthesized compounds were individually docked to selected regions of proteins. The results are presented in Table 7. The docking scores of known cancer-related drugs were also examined for comparison. Erlotinib, sorafenib, and copanlisib were optimized for targeting EGFR, VEGFR2, and PI3K, respectively. These 3 protein-ligand pairings had lower docking scores than the synthesized compounds in the current study. Conversely, FAK-ifebemtinib has a similar docking score to the synthesized compounds in the current study.

Docking scores and MMPBSA values were analyzed together to evaluate the binding affinity and binding stability of the ligands. Docking scores and MMPBSA calculations differ in their purpose, scope, and depth of analysis they provide. MMPBSA is more accurate as it considers dynamic and solvent effects, whereas docking scores are simpler and less detailed. Docking scores are mainly for ranking and screening, while MMPBSA provides insights into binding thermodynamics. Thus, docking can be used for initial screening, and MMPBSA can refine and validate the binding affinities of promising ligand-receptor complexes [81].

The docking scores for all compounds ranged between −8.8 and −11.0 kcal/mol, indicating strong binding affinity with the target proteins. While compound 6 had a high binding affinity with the 1M17 and 2XIR targets, the MMPBSA value for 2XIR (−6.30 kcal/mol) indicates relatively lower binding stability. However, compound 6 had strong and stable binding with the 1E8X (−71.16 kcal/mol) and 1MP8 (−55.29 kcal/mol) targets. Although compound 7a had consistently low docking scores (high binding affinity) across all targets, it had the most negative MMPBSA value with the 1E8X target (−108.81 kcal/mol), indicating a highly stable binding complex with this protein. Compound 7b had high binding stability, particularly with the 1E8X (−77.28 kcal/mol) and 1MP8 (−79.20 kcal/mol) targets. This suggests a strong binding potential with these 2 proteins. Docking scores of compound 7c with the selected proteins were higher (weaker binding affinity) compared to the other compounds, and it showed a particularly low MMPBSA value with the 2XIR target (−1.58 kcal/mol). This indicates weaker binding stability compared to the other compounds. Compound 7d performed well in terms of docking scores and MMPBSA values. It formed highly stable complexes, particularly with the 1M17 (−80.51 kcal/mol), 1E8X (−80.07 kcal/mol), and 1MP8 (−76.56 kcal/mol) targets.

The data presented in Table 7 highlight the differences in binding affinity and stability among the compounds. Among the compounds, 7a emerged as the most stable candidate with the 1E8X target. Compound 7d had stable binding with multiple targets, making it a versatile candidate. In contrast, compound 7c had the weakest binding stability among the evaluated compounds. This analysis suggests that the identified lead compounds, particularly 7a and 7d, should be further validated experimentally to confirm their biological activity and therapeutic potential.

1M17-7d, 2XIR-7b, 1E8X-7d, and 1MP8-7b are the complex pairs with the best docking score and were therefore selected for 2D interaction assessment.

**Figure 2:**
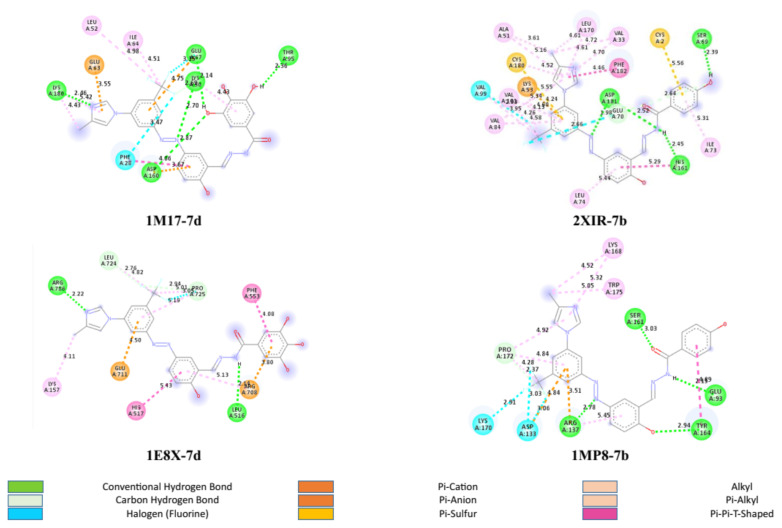
2D representation of protein-ligand interactions.

Among the 1M17-ligand complexes, the 1M17-7d complex had the best docking score. When the 1M17-7d complex was examined, there were various interactions including hydrogen bond, carbon-hydrogen bond, halogen, π-anion, π-π stacked, alkyl, and π-alkyl interactions (Figure 2). Since hydrogen is attached to an atom that is relatively electronegative and acts as a hydrogen donor, hydrogen bonds are the strongest kind of bonds that can form between molecules. There were 5 hydrogen bonds between the 1M17 protein and the 7d ligand. These bonds were between LYS180 and the nitrogen atom of imidazol with 2.46 Å bond length, GLU67 and phenol hydrogen with 2.14 Å bond length, LYS50 and the nitrogen of the azo group with 2.70 Å bond length, THR95 and phenol hydrogen at 2.36 Å bond length, and ASP160 and phenol oxygen with 2.87 Å bond length. The presence of 5 hydrogen bonds in the 1M17-7d complex suggests that the structure is more stable compared to the other 1M17-ligand complexes.

Regarding the 2XIR-ligand complexes, 2XIR-7b and 2XIR-7d complexes have the best docking scores. As compound 7b has a higher cytotoxic activity than compound 7d, interactions between 2XIR and 7b complex were examined in more detail. Figure 2 shows there were various interactions between 2XIR and 7b, including hydrogen bond, carbon-hydrogen bond, halogen, π-cation, π-sulfur, π-π stacked, π-π T-shaped, alkyl, and π-alkyl interactions. There were 4 hydrogen bonds in the 2XIR-7b complex. These bonds were between ASP181 and the nitrogen atom of the azo group with 1.98 Å bond length, ASP181 and nitrogen of hydrazide with 2.52 Å bond length, HIS161 and nitrogen of hydrazide with 2.45 Å bond length, and SER69 and phenol hydrogen with 2.39 Å bond length.

The IE8X-7d complex had a docking score of −10.1 kcal/mol, followed closely by IE8X-7b with 10 kcal/mol. Therefore, the 1E8X-7d complex was chosen for examining 2D interactions. Figure 2 shows that there were various interactions between IE8X and 7d, including hydrogen bond, carbon-hydrogen bond, halogen, π-cation, π-anion, π-π stacked, alkyl, and π-alkyl interactions. There were 2 hydrogen bonds in the 1E8X-7d complex. These bonds were between ARG786 and the nitrogen atom of imidazole with a 2.22 Å bond length, and LEU516 and the hydrogen atom of hydrazide with a 2.14 Å bond length.

1MP8-7a, 1MP8-7b, and 1MP8-7d complexes had the same docking score of −9.2 kcal/mol. As the 7b ligand had a higher cytotoxic activity than 7d for both MCF-7 and A549, and a higher cytotoxic activity than 7a for A549, the 1MP8-7b complex was chosen to examine 2D interactions. Figure 2 shows that there were various interactions between IMP8 and 7b, including hydrogen bond, carbon-hydrogen bond, halogen, π-cation, π-anion, π-π T-shaped, alkyl, and π-alkyl interactions. Four hydrogen bonds were between ARG137 and the nitrogen atom of the azo group with a 2.78 Å bond length, TYR164 and the phenol oxygen atom with a 2.94 Å bond length, GLU93 and the hydrogen atom of hydrazide with a 2.15 Å bond length, and SER161 and the oxygen atom of the carbonyl group with a 3.03 Å bond length.

#### 3.4.3. Molecular dynamic simulation

**Figure 3:**
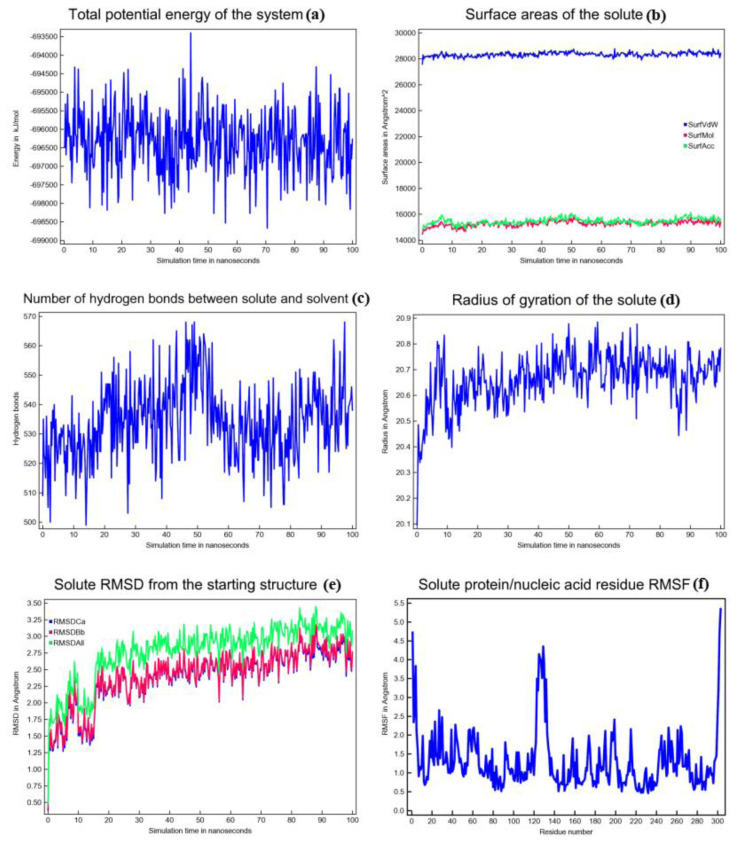
(a) The total potential energy of the system, (b) SASA, (c) hydrogen bonds, (d) Rg, (e) RMSD, and (f) RMSF plots for the 100 ns MD simulation of the 2XIR-7c complex.

Although compound 7c had the weakest binding stability among the evaluated compounds, it had greater cytotoxic activity against the MCF-7 cancer cell line compared to the others. Additionally, the 2XIR-compound complexes showed higher docking scores than other proteins. Therefore, an MD simulation study was conducted to further assess the stability of 2XIR-7c. The results related MD simulation studies were illustrated in Figures 3a, 3b, 3c, 3d, 3e and 3f, respectively.

Figure 3a depicts the variation in the total potential energy of the system over a 100 ns simulation. The total potential energy fluctuates within a specific range, approximately between −693,500 and −698,500 kJ/mol. Despite these oscillations, the energy remains confined to this range throughout the simulation. Such fluctuations are characteristic of MD simulations and arise from the thermal motion and dynamic interactions of particles within the system. The stability of the energy values over time suggests that the system has reached an equilibrium state and maintained it throughout the simulation. This indicates that the simulation parameters, such as temperature, pressure, and integration settings, were appropriately chosen to allow the system to achieve and sustain a thermodynamically stable state.

Figure 3b illustrates the temporal evolution of the surface areas of the solute (SASA) throughout a 100 ns MD simulation. The graph displays 3 distinct types of surface areas. SurfVdW (blue curve) represents the van der Waals surface area of the solute. It remains relatively stable throughout the simulation, fluctuating within a narrow range of around 28,000 Å^2^. The constancy of this value indicates minimal structural changes in the solute at the van der Waals level, suggesting that the solute maintains its conformational integrity during the simulation. SurfMol (molecular surface area, red curve) is slightly smaller than the van der Waals surface area, remaining stable at around 15,000 Å^2^. The small fluctuations around this value indicate the molecular surface of the solute is consistently exposed to its environment and there are minimal alterations in the overall geometry and flexibility. SurfAcc (solvent-accessible surface area, green curve) represents the solvent-accessible surface area that fluctuates around 15,000 Å^2^, similar to the SurfMol. The solvent-accessible surface reflects the regions of the solute accessible to the solvent molecules. The stability of this parameter indicates that the solute maintains a consistent degree of solvent exposure, implying no significant folding, unfolding, or conformational rearrangements during the simulation. Figure 3b suggests that the surface properties of the solute are stable over the simulation period, indicating a lack of large-scale conformational changes or significant interactions that might alter its surface exposure. This stability is critical for understanding the behavior of the solute in its simulated environment, including its interactions with other molecules or solvents.

Figure 3c illustrates the temporal variation in the number of hydrogen bonds between the solute and its surrounding solvent over a 100 ns MD simulation. The number of hydrogen bonds fluctuates between approximately 500 and 570 during the simulation. These fluctuations are expected due to the dynamic nature of molecular interactions in a solvent environment. Thermal motion, conformational changes of the solute, and solvent rearrangement contribute to the transient formation and breaking of hydrogen bonds. Despite the fluctuations, the number of hydrogen bonds remains within a relatively narrow range, indicating that the solute-solvent interactions are stable over time. This suggests the hydrogen bonding capability of the solute with the solvent remains consistent throughout the simulation.

Monitoring the radius of gyration (Rg) in MD simulations is important for understanding the structural dynamics of molecules over time. Rg allows for tracking structural changes and transitions (folding, unfolding, aggregation), assessing the equilibrium and stability of the system, characterizing the size, shape, and compactness of molecules or complexes. This provides a reliable basis to validate simulation results and compare them with experimental data, offering insights into molecular interactions, flexibility, and response to environmental factors. Figure 3d displays Rg values over time. At the beginning, the Rg value of the complex was 20.4 Å. Average Rg value of the complex during the simulation 20.7 Å. At the end of the 100 ns simulation, the Rg value of the complex was also 20.7 Å. This means that the structure of the 2XIR-7c complex remained stable during 100 ns and did not show any notable conformational changes.

**Figure 4:**
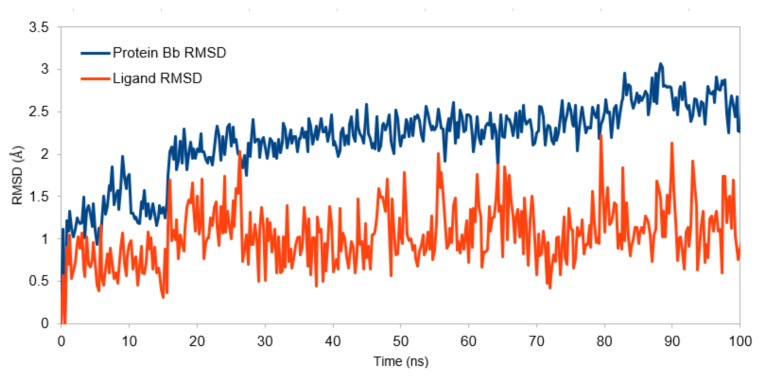
RMSD graph for the protein backbone (Bb) and ligand.

The root mean square deviation (RMSD) is a common metric to assess the stability and conformational changes of a molecular system over time. Figure 3e illustrates RMSD of the atomic positions of the solute from its starting structure during the MD simulation. RMSDCα represents the RMSD of the alpha carbon atoms in the 2XIR protein. Alpha carbons are often used to monitor the overall stability of the protein backbone. RMSDBb represents the RMSD of the entire backbone of the protein. RMSDAll reflects the RMSD of all atoms in the system, including side chains and possibly solvent molecules. Initially, all curves start at a low RMSD (close to 0), as the system starts in its reference structure. As the simulation progresses, the RMSD values increase, reflecting structural deviations from the starting configuration. After approximately 20–30 ns, the curves appear to stabilize, suggesting the system has reached equilibrium and fluctuations become more uniform. The stabilization of RMSD values after approximately 20–30 ns indicates that the system has reached a relatively stable conformation. RMSDAll has the highest RMSD values, as it includes side chain and possibly solvent motions, which are more flexible than the backbone. RMSDBb and RMSDCα curves show lower RMSD values, indicating the backbone, particularly the alpha carbons, remains relatively stable compared to the entire structure. The RMSD graph of the 7c ligand during 100 ns simulation was also obtained from the MD simulation calculations and illustrated in Figure 4. RMSD values of the ligand fluctuate greatly between approximately 0.5 and 2 Å. This variability suggests that the ligand undergoes dynamic conformational changes within the binding pocket. However, the absence of prolonged deviations beyond 2.5 Å suggests that the ligand remains associated with the protein, albeit with some degree of flexibility.

The root mean square fluctuation (RMSF) term in MD simulations refers to the amount that individual atoms or groups of atoms move or fluctuate about their average position during the simulation. It is taken as the root mean square of the difference between the average position of each atom and their positions at each time step to compute RMSF. RMSF values provide an insight into how stable or flexible various molecular constituents are. Higher RMSF levels correspond to more mobility or flexibility, while lower RMSF values correspond to increased stiffness or stability. RMSF values of the protein are displayed in Figure 3f. RMSF values were mostly below 3 Å throughout the simulation, indicating relative stability in the complex. Although, there are some minor fluctuations at residue number locations 125–135. This means that the protein in the structure of the complex changed very little except for these amino acid residues.

**Figure 5:**
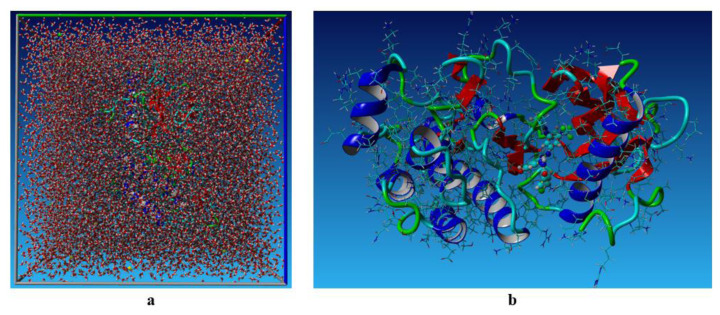
A snapshot of the last simulation scene of the 2XIR-7c complex. (a) Solvent, protein, and ligand; (b) only the protein and ligand without the solvent.

The images in Figure 5 represent the final snapshot (at 100 ns) of the MD simulation of the 2XIR-7c complex, illustrating different levels of system composition. Figure 5a shows the entire simulation box containing the protein-ligand complex fully solvated in water. The protein structure (green/blue) and ligand (small molecule) are embedded within the solvent. The solvent molecules and simulation box are removed to provide a clearer view of the molecular interactions in Figure 5b. The secondary structure of the protein (alpha helices in blue, beta sheets in red) are visible, along with the ligand bound to the protein.

## 4. Conclusions

In this study, 5 novel imidazole-bearing hydrazone compounds were synthesized and characterized using spectroscopic techniques such as FTIR, ^1^H-NMR, ^13^C-NMR, and MS. The absorption properties of compounds in DMSO were evaluated. ADMET properties were assessed using the ADMETlab 2.0 web server. From the ADMET results, it was concluded that 6, 7a, and 7c drug candidates could be used as drugs after further investigations were performed. The cytotoxic activity properties of compounds were investigated against cells lines representing the most common cancer types: breast (MCF-7) and lung (A549). The IC_50_ values of tested compounds were lower than 10 μM, except for compound 7d in A549 (IC_50_ = 29.07 μM). In the MCF-7 cell line, all compounds had more cytotoxic activity (approximately 10×) than the positive control drug cisplatin. The thiophene-cored compound 7c had the highest cytotoxic activity with a 0.6312 μM IC_50_ value in MCF-7. Compound 7b had the most cytotoxic activity with a 3.472 μM IC_50_ value, which was close to cisplatin, in A549. Despite having the lowest cytotoxic activity in both MCF-7 and A549, compound 7d still had high cytotoxic activity against MCF-7 than cisplatin. The molecular docking analyses were also conducted for synthesized compounds with cancer-related proteins 1M17, 2XIR, 1E8X, and 1MP8. The docking scores were between −8.8 and −11.0 kcal/mol. The 2XIR-7b complex had the highest score with 11.0 kcal/mol, while 1MP8-6 had the lowest score with 8.8 kcal/mol. An MD simulation study was also performed to support the stability of the 2XIR-7c complex. The MD simulation results showed that the complex was stable during the 100 ns simulation. The compound that holds the greatest promise for further investigation in related fields such as in vivo experiments is 7c. These results will help researchers working on anticancer agents.

## Supplementary Information



## Figures and Tables

**Table 1:** Binding site coordinates of the selected proteins.

PDB ID	X	Y	Z
**1M17**	24.8	2.1	51.0
**2XIR**	21.0	26.2	38.8
**1E8X**	20.2	64.8	21.8
**1MP8**	38.1	−3.6	23.8

**Table 2:** Synthesis of esters 2a and 2c, and hydrazide compounds 3a–3d.

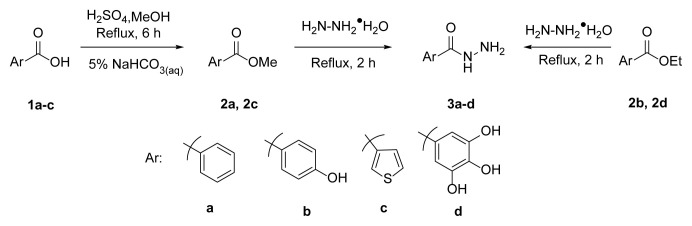		
Substrate	Ester (Yield%)	Hydrazide (Yield%)
1a	2a (95)	3a (96)
-	2b	3b (92)
1c	2c (92)	3c (88)
-	2d	3d (80)

**Table 3:** Synthesis of imidazole-bearing hydrazone derivatives (6 and 7a–7d).

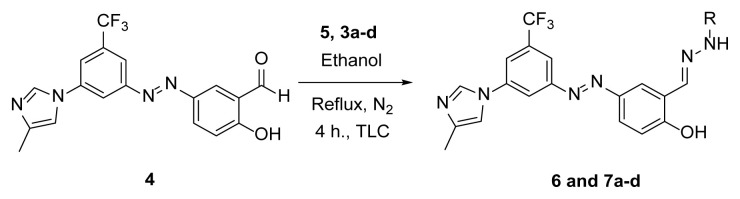				
	Substrate		Product	Yield (%)
**5**	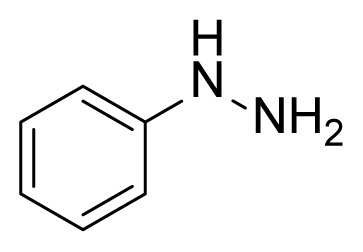	**6**	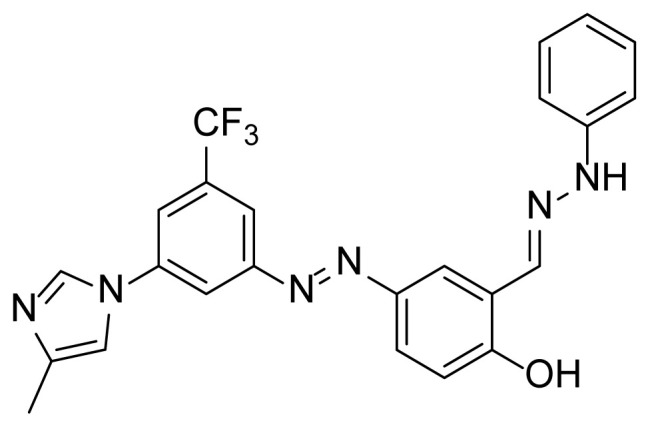	76
**3a**	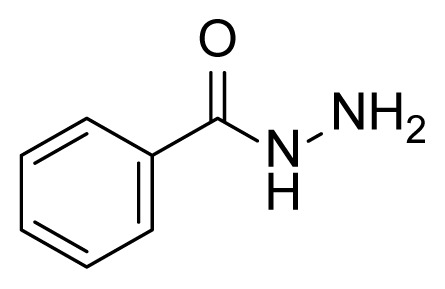	**7a**	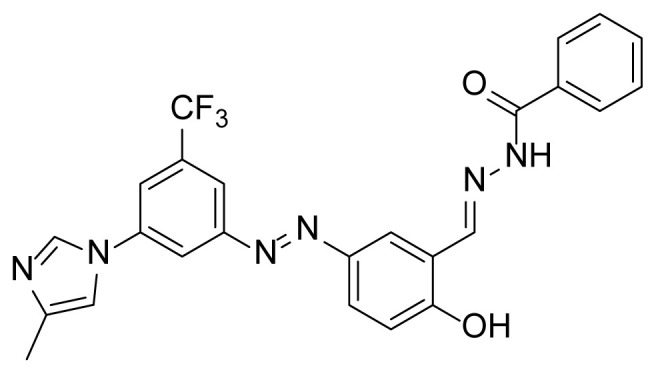	81
**3b**	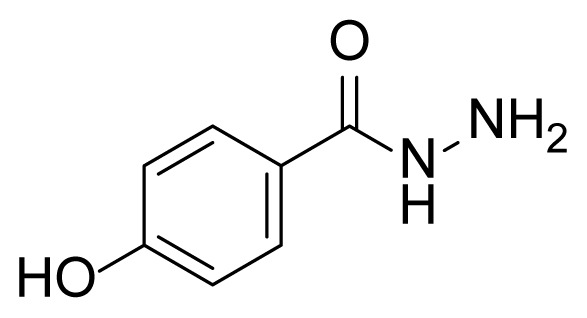	**7b**	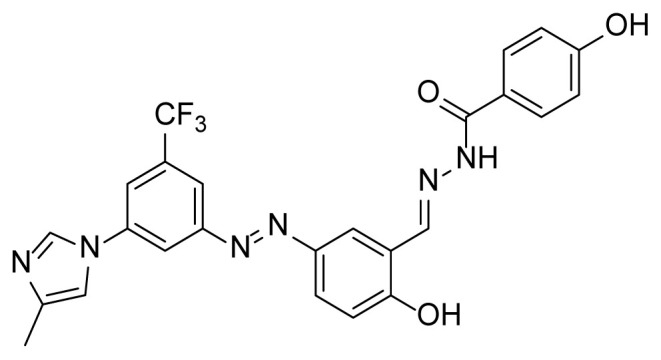	70
**3c**	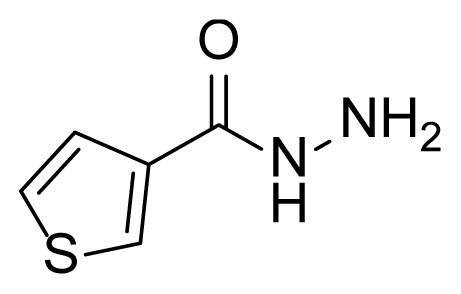	**7c**	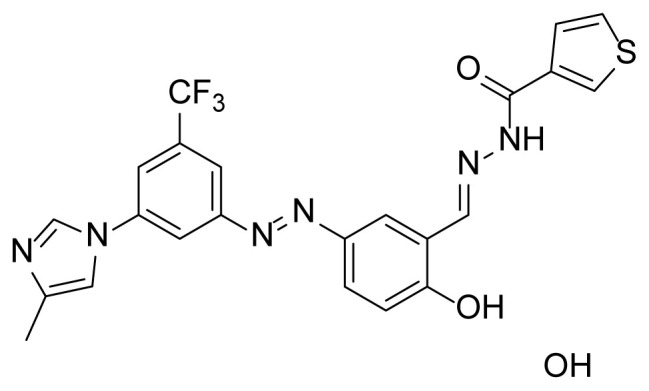	83
**3d**	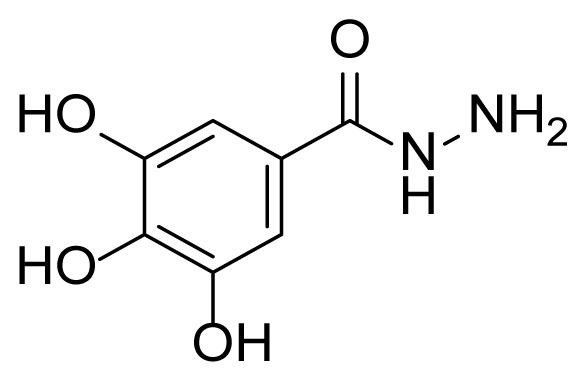	**7d**	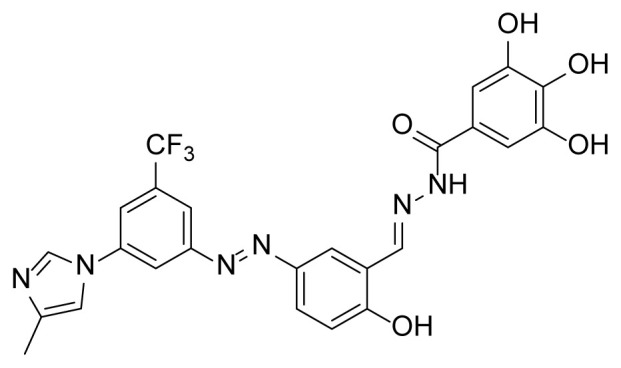	74

**Table 4:** Maximum wavelengths of synthesized compounds.

Compounds	Maximum Wavelengths (nm)
6	λ1 = 263, λ2 = 357, λ3 = 445
7a	λ1 = 263, λ2 = 319, λ3 = 375, λ4 = 518
7b	λ1 = 263, λ2 = 323, λ3 = 375
7c	λ1 = 263, λ2 = 319, λ3 = 375, λ4 = 525
7d	λ1 = 263, λ2 = 327, λ3 = 379

**Table 5:** Cytotoxicity activity results of compounds 6 and 7a–7d against MCF-7 and A549 cell lines.

Compounds	IC_50_ (μM)*	
	
	MCF-7	A549
	
**6**	0.6644	5.580
**7a**	0.6402	4.499
**7b**	0.7230	3.472
**7c**	0.6312	4.056
**7d**	0.8203	29.07
Cisplatin	9.262	2.605

* Concentration required to achieve 50% cell inhibition.

**Table 6:** Calculated physicochemical, medicinal, and ADMET parameters of compounds 6 and 7a–7d.

Parameters		6	7a	7b	7c	7d	Recommended Values
**Physicochemical Property**	Molecular Weight (g/mol)	464.16	492.15	508.15	498.11	540.14	0–500
	TPSA (Å^2^)	87.16	104.23	124.46	104.23	164.92	0–140
**Medicinal Chemistry**	Lipinski Rule	Accepted	Accepted	Rejected	Accepted	Rejected	Accepted
**Absorption**	**Caco-2 Permeability**	−4.936	−4.966	−5.028	−5.143	−5.449	>−5.15
	**MDCK Permeability**	1.2 × 10^−5^	8 × 10^−6^	5 × 10^−6^	2.3 × 10^−5^	5 × 10^−6^	>20 × 10^−6^
**Distribution**	**PPB**	101.6%	100.2%	100%	100%	100%	<90%
	**BBB Penetration**	0.005	0.005	0.004	0.011	0.002	0–0.3: excellent 0.3–0.7: medium 0.7–1.0: poor
**Metabolism**	**CYP1A2 inhibitor**	0.954	0.73	0.666	0.8	0.696	Category 0: Nonsubstrate / Noninhibitor; Category 1: substrate / inhibitor
	**CYP1A2 substrate**	0.537	0.454	0.224	0.589	0.338	
	**CYP2C19 inhibitor**	0.952	0.883	0.693	0.864	0.2	
	**CYP2C19 substrate**	0.058	0.059	0.051	0.057	0.042	
	**CYP2C9 inhibitor**	0.716	0.906	0.831	0.761	0.649	
	**CYP2C9 substrate**	0.259	0.223	0.33	0.269	0.065	
	**CYP2D6 inhibitor**	0.392	0.417	0.225	0.092	0.046	
	**CYP2D6 substrate**	0.387	0.164	0.207	0.261	0.155	
	**CYP3A4 inhibitor**	0.748	0.786	0.834	0.79	0.668	
	**CYP3A4 substrate**	0.293	0.234	0.219	0.319	0.12	
**Excretion**	**CL**	4.822	3.519	4.572	3.131	5.359	High: >15 mL/min/kg; moderate: 5–15 mL/min/kg; low: <5 mL/min/kg
	**T ** **_1/2_**	0.127	0.161	0.309	0.121	0.766	0–0.3: excellent 0.3–0.7: medium 0.7–1.0: poor
**Toxicity**	**hERG Blockers**	0.662	0.626	0.554	0.738	0.236	
	**AMES Toxicity**	0.195	0.666	0.569	0.708	0.344	
	**Rat Oral Acute Toxicity**	0.027	0.042	0.079	0.043	0.314	
	LD50 (mg/kg)	1000	125	125	125	125	
	Toxicity class	4	3	3	3	3	Worst to best 1 to 6

**Table 7:** Molecular docking parameters for the ligand-target molecule pairs.

	Selected proteins			
Compound	Docking score, kcal/mol (MM/PBSA, kcal/mol)			
	1M17	2XIR	1E8X	1MP8
**6**	−9.7 (−34.18)	−10.5 (−6.30)	−9.9 (−71.16)	−8.8 (−55.29)
**7a**	−9.7 (−24.94)	−10.9 (−10.76)	−9.9 (−108.81)	−9.2 (−69.59)
**7b**	−9.7 (−49.93)	−11.0 (−8.45)	−10.0 (−77.28)	−9.2 (−79.20)
**7c**	−9.1 (−25.13)	−10.6 (−1.58)	−9.3 (−54.07)	−9.0 (−54.39)
**7d**	−9.8 (−80.51)	−11.0 (−26.98)	−10.1 (−80.07)	−9.2 (−76.56)
**Erlotinib**	−6.8 (−43.43)	-	-	-
**Sorafenib**	-	−10.0 (−16.97)	-	-
**Copanlisib**	-	-	−8.3 (−99.90)	-
**Ifebemtinib**	-	-	-	−9.2 (−106.29)
