# Supplementary material for: Comprehensive study of imidazole-based hydrazones: From design, synthesis, and characterization to in vitro and in silico antiproliferative activity analysis
Source: Turk J Chem. 2025 Apr 15;49(4):419–38. doi: 10.55730/1300-0527.3741 (PMC13052440; doi:10.55730/1300-0527.3741)
Supplement: Supplementary file 1 [file SupportingInformation_21052025.docx]

Supporting Information for

**Comprehensive Study of Imidazole-Based Hydrazones: From Design, Synthesis, and Characterization to *In Vitro* and *In Silico* Antiproliferative Activity Analysis**

Ömer DİLEK

*Isparta University of Applied Sciences, Central Research Laboratory Application and Research Center, 32200, Isparta, Türkiye*

*E-mail:* [*omerdilek@isparta.edu.tr*](mailto:omerdilek@isparta.edu.tr)

**Table of Contents**

| **Page(s)** | **Content** |
| --- | --- |
| **S1–S2** | Table of Contents |
| **S3–S10** | NMR Spectra |
| **S3** | ^1^H NMR Spectrum of **6** (700 MHz, DMSO-*d_6_*) |
| **S3** | ^13^C{^1^H} NMR Spectrum of **6** (APT, 176 MHz, DMSO-*d_6_*) |
| **S4** | ^1^H NMR Spectrum of **7a** (400 MHz, DMSO-*d_6_*) |
| **S4-S5** | ^13^C{^1^H} NMR Spectrum of **7a** (APT, 101 MHz, DMSO-*d_6_*) |
| **S6** | ^1^H NMR Spectrum of **7b** (400 MHz, DMSO-*d_6_*) |
| **S6-S7** | ^13^C{^1^H} NMR Spectrum of **7b** (APT, 101 MHz, DMSO-*d_6_*) |
| **S7** | ^1^H NMR Spectrum of **7c** (400 MHz, DMSO-*d_6_*) |
| **S8** | ^13^C{^1^H} NMR Spectrum of **7c** (APT, 101 MHz, DMSO-*d_6_*) |
| **S9** | ^1^H NMR Spectrum of **7d** (400 MHz, DMSO-*d_6_*) |
| **S9-S10** | ^13^C{^1^H} NMR Spectrum of **7d** (APT, 101 MHz, DMSO-*d_6_*) |
| **S11-20** | Mass Spectra |
| **S11** | Mass Spectrum of **6** (Positive Mode) |
| **S12** | Mass Spectrum of **6** (Negative Mode) |
| **S13** | Mass Spectrum of **7a** (Positive Mode) |
| **S14** | Mass Spectrum of **7a** (Negative Mode) |
| **S15** | Mass Spectrum of **7b** (Positive Mode) |
| **S16** | Mass Spectrum of **7b** (Negative Mode) |
| **S17** | Mass Spectrum of **7c** (Positive Mode) |
| **S18** | Mass Spectrum of **7c** (Negative Mode) |
| **S19** | Mass Spectrum of **7d** (Positive Mode) |
| **S20-22** | FTIR Spectra |
| **S20** | FTIR Spectra of **6** |
| **S20** | FTIR Spectra of **7a** |
| **S21** | FTIR Spectra of **7b** |
| **S21** | FTIR Spectra of **7c** |
| **S22** | FTIR Spectra of **7d** |

**NMR Spectra**

^1^H-NMR Spectrum of **6** (700 MHz, DMSO-d_6_)

^13^C{^1^H} NMR Spectrum of **6** (APT, 176 Mhz, DMSO-d_6_)

^1^H-NMR Spectrum of **7a** (400 MHz, DMSO-d_6_)


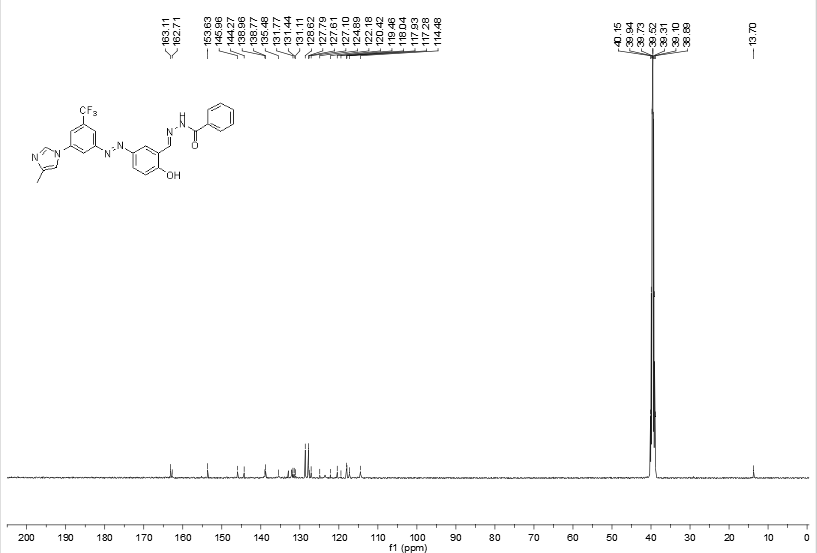


^13^C{^1^H} NMR Spectrum of **7a** (APT, 101 MHz, DMSO-d_6_)


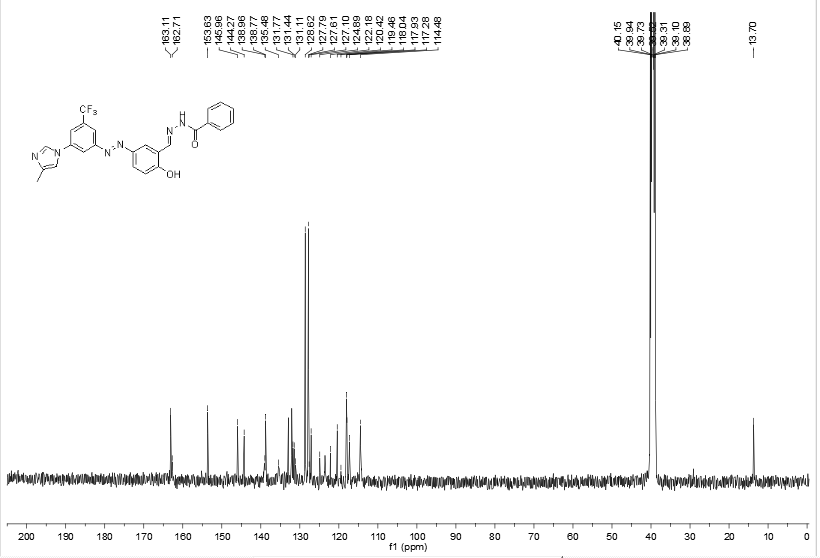


^13^C{^1^H} NMR Spectrum of **7a** (APT, 101 MHz, DMSO-d_6_)


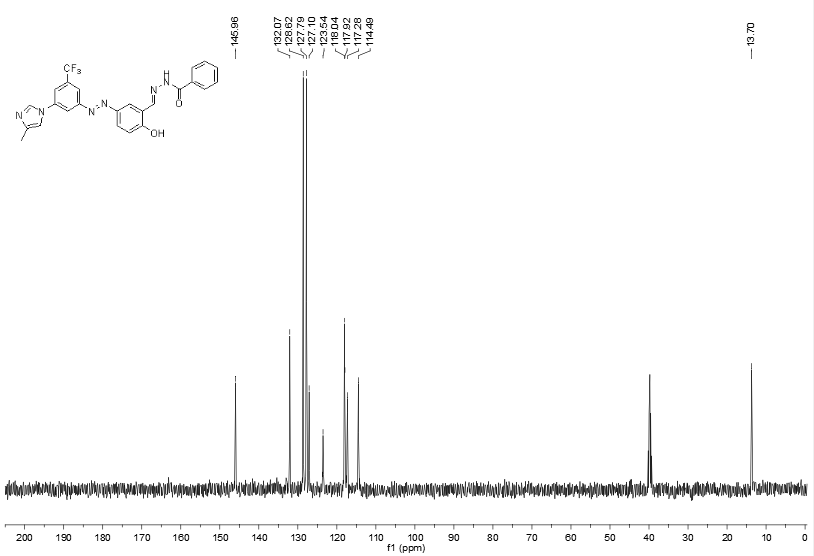


^13^C{^1^H}-DEPT NMR Spectrum of **7a** (APT, 101 MHz, DMSO-d_6_)

^1^H-NMR Spectrum of **7b** (400 MHz, DMSO-d_6_)


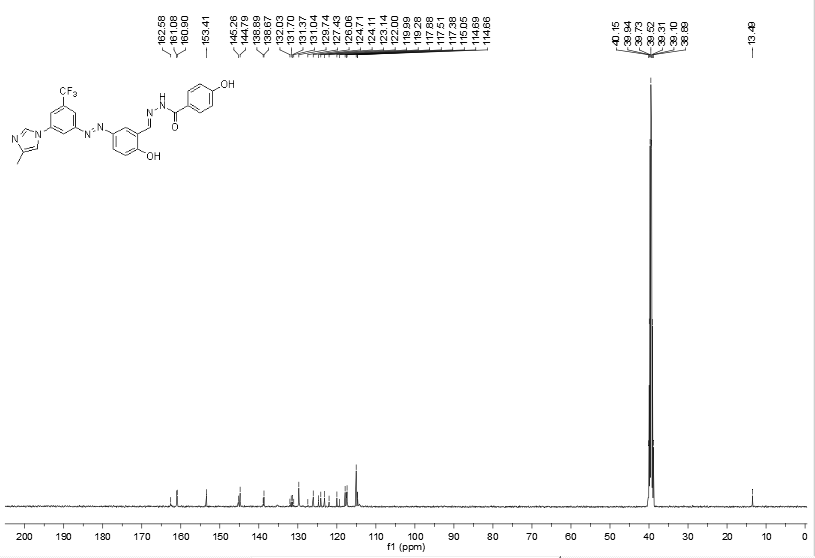


^13^C{^1^H} NMR Spectrum of **7b** (APT, 101 MHz, DMSO-d_6_)


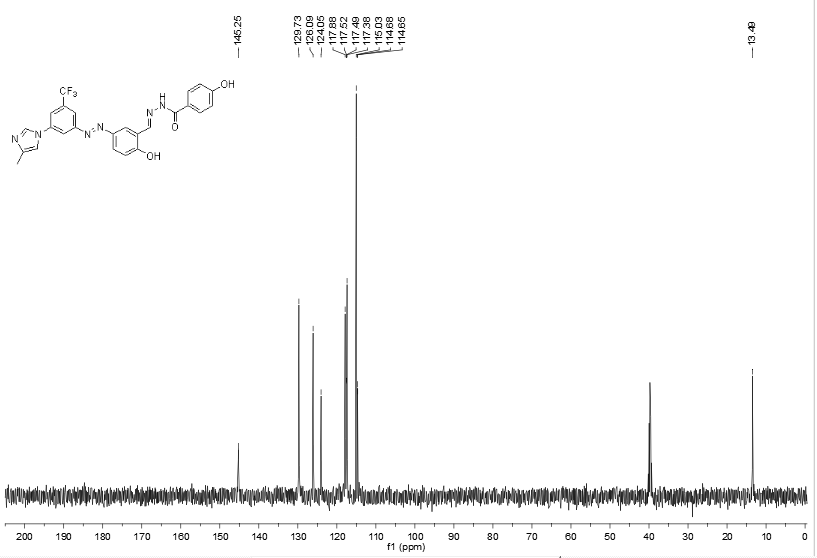


^13^C{^1^H}-DEPT NMR Spectrum of **7b** (APT, 101 MHz, DMSO-d_6_)

^1^H-NMR Spectrum of **7c** (400 MHz, DMSO-*d_6_*)


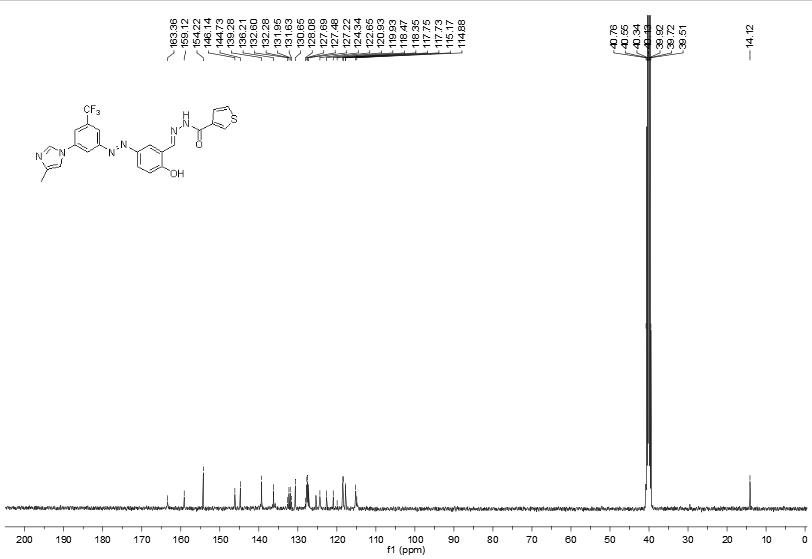


^13^C{^1^H} NMR Spectrum of **7c** (APT, 101 MHz, DMSO-d_6_)


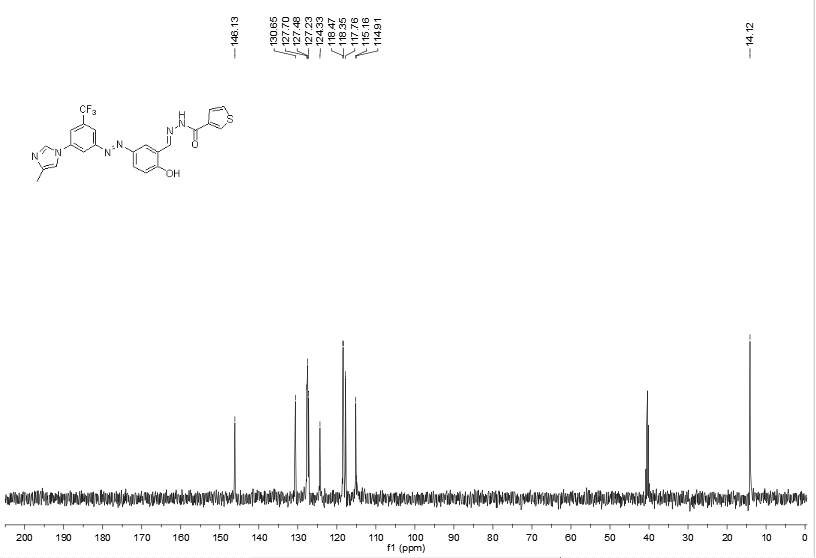


^13^C{^1^H}-DEPT NMR Spectrum of **7c** (APT, 101 MHz, DMSO-d_6_)


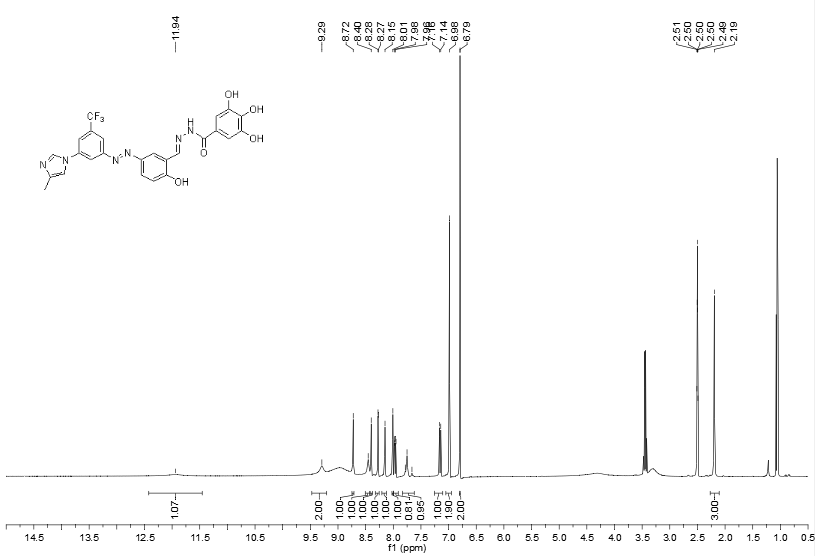


Ethanol solvent residue

^1^H-NMR Spectrum of **7d** (400 MHz, DMSO-*d_6_*)


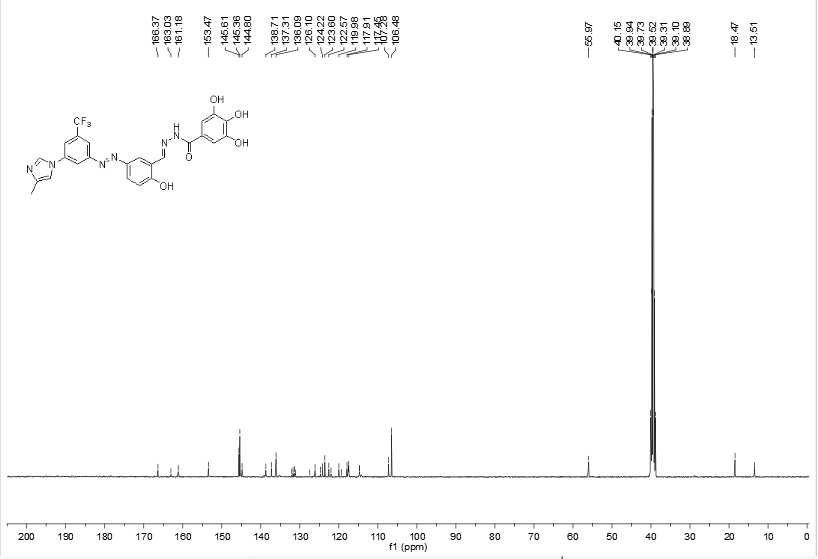


Ethanol solvent residue

^13^C{^1^H} NMR Spectrum of **7d** (APT, 101 MHz, DMSO-d_6_)


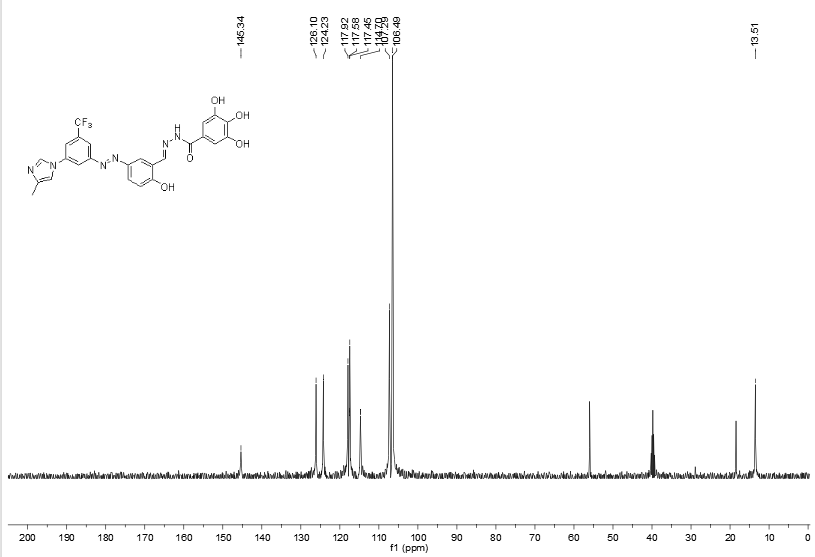


Ethanol solvent residue

^13^C{^1^H}-DEPT NMR Spectrum of **7d** (APT, 101 MHz, DMSO-d_6_)

**Mass Spectra**


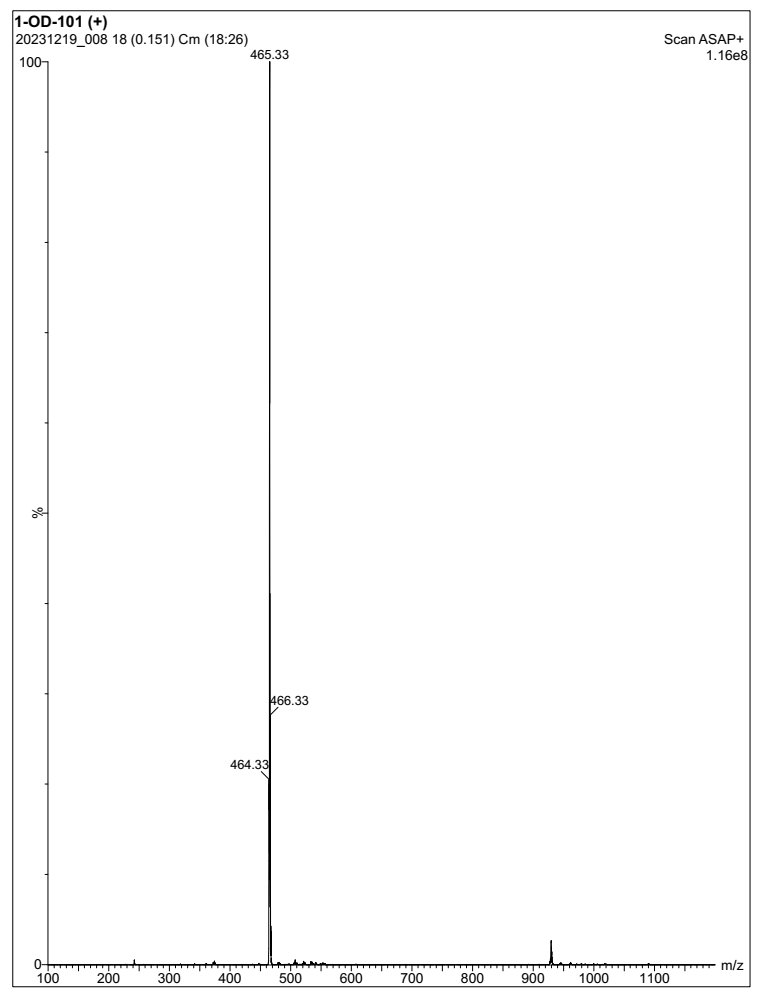

Mass Spectrum of compound **6** (Positive Mode)


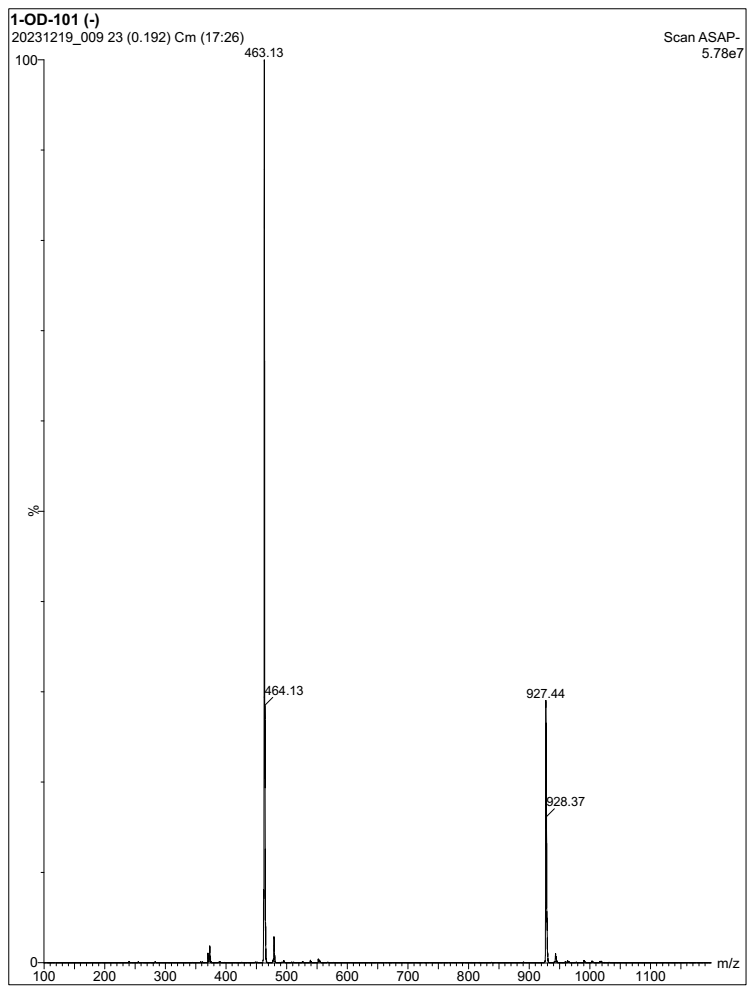

Mass Spectrum of compound **6** (Negative Mode)


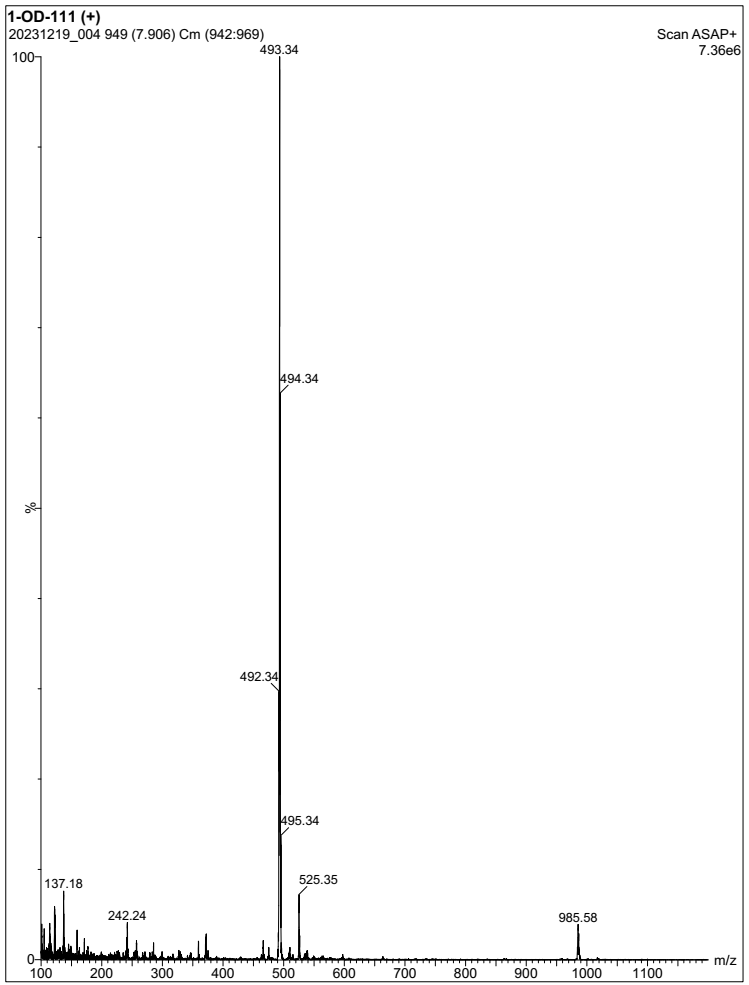

Mass Spectrum of compound **7a** (Positive Mode)


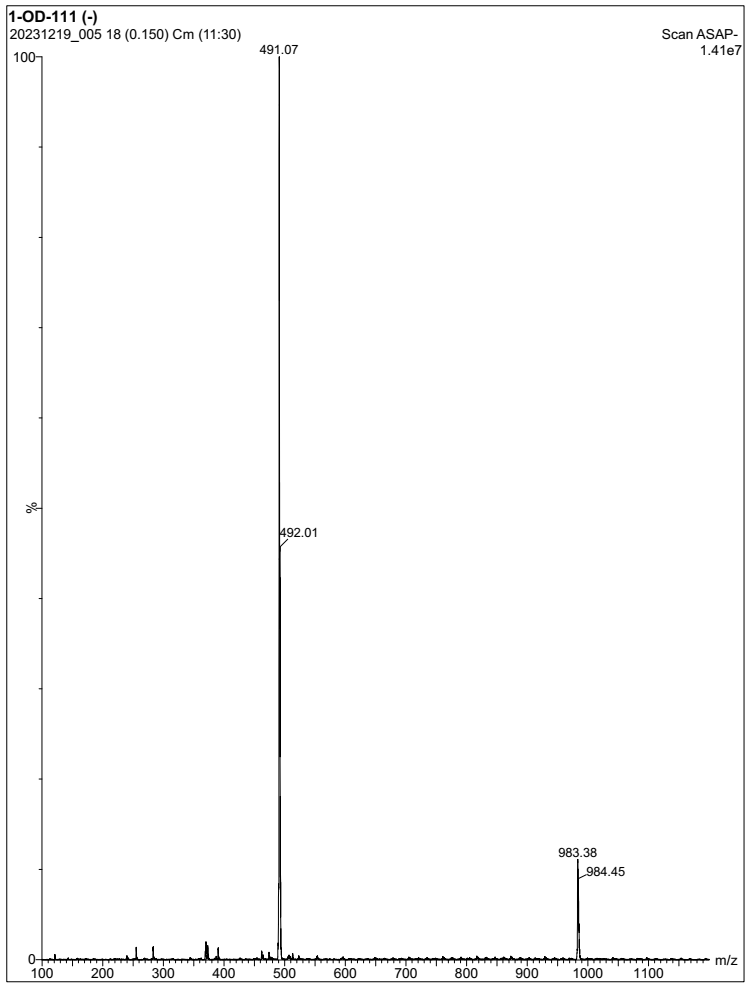

Mass Spectrum of compound **7a** (Negative Mode)


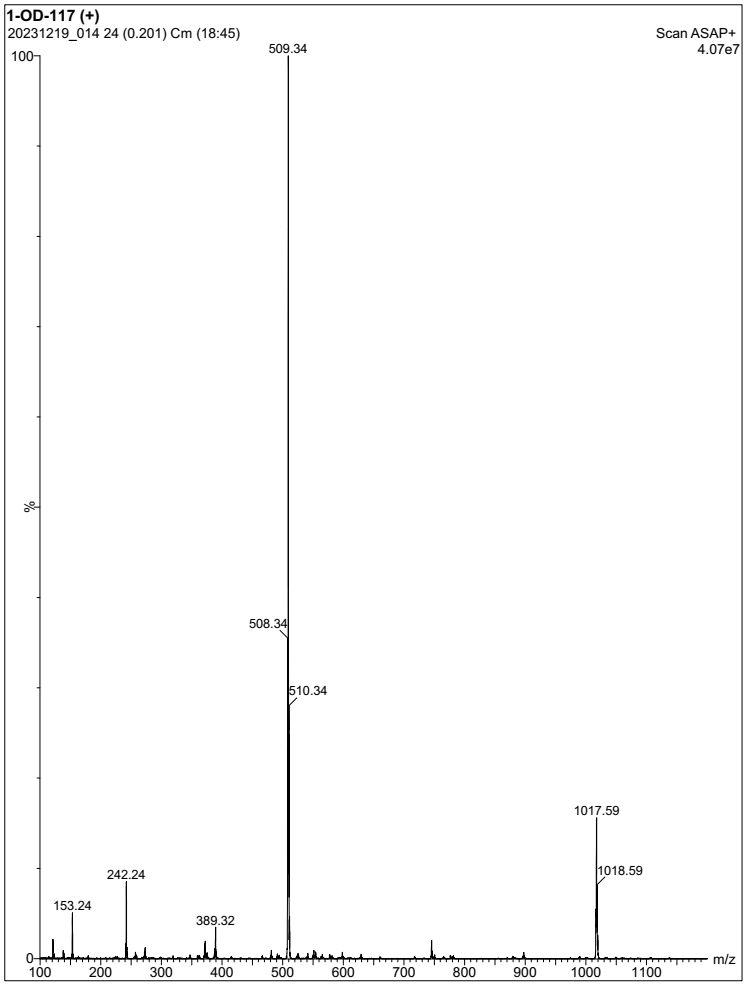

Mass Spectrum of compound **7b** (Positive Mode)


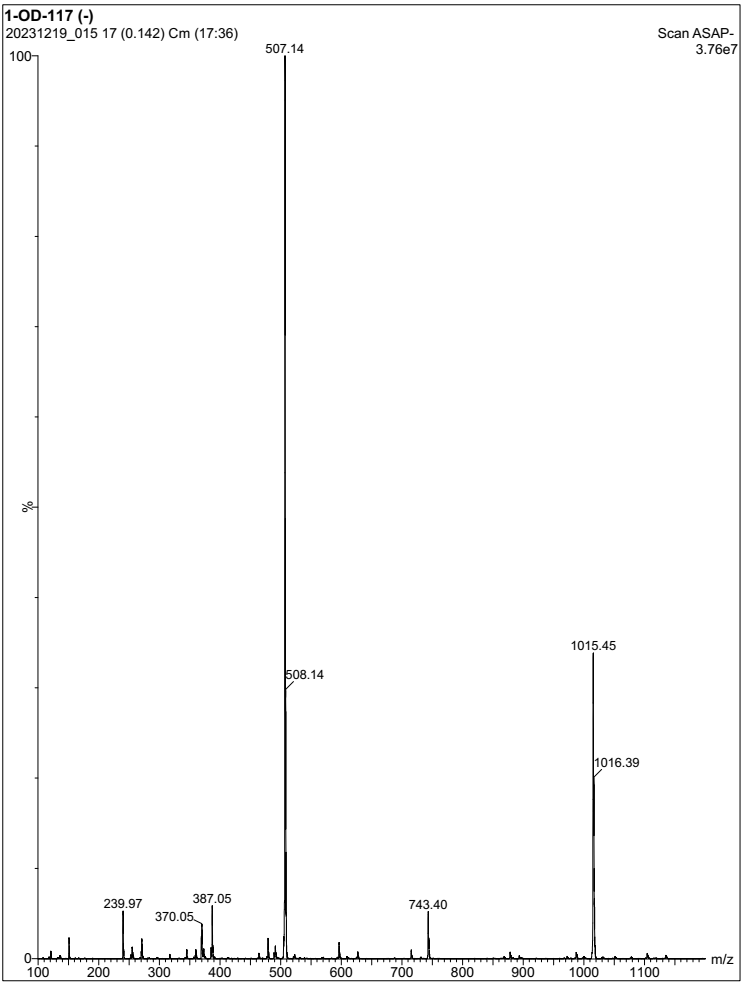

Mass Spectrum of compound **7b** (Negative Mode)


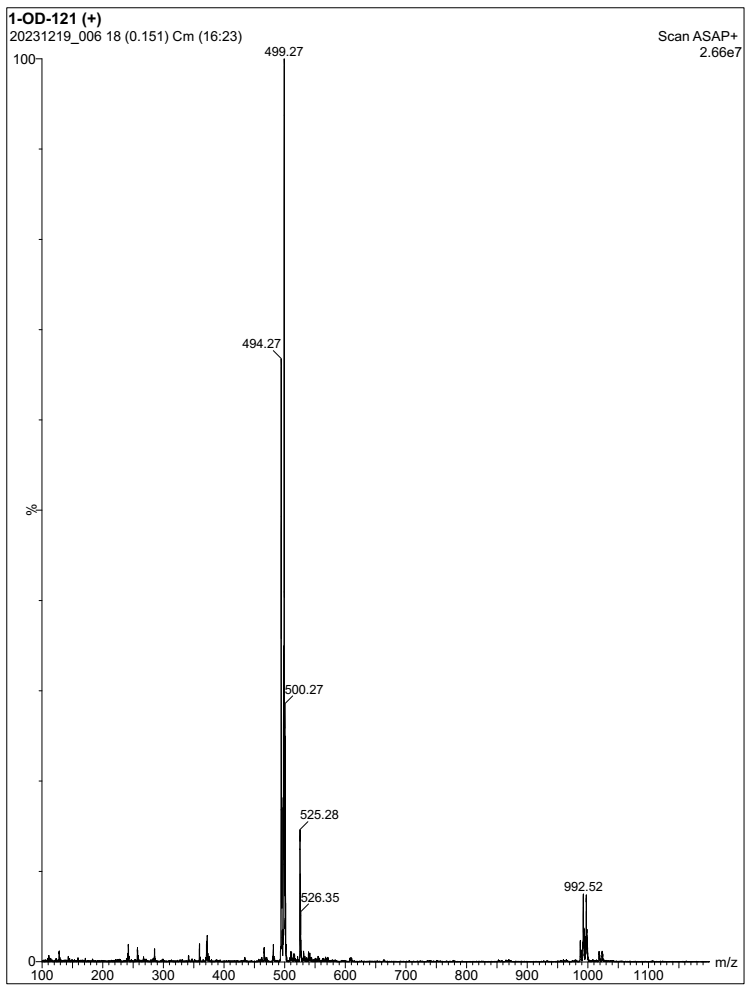

Mass Spectrum of compound **7c** (Positive Mode)


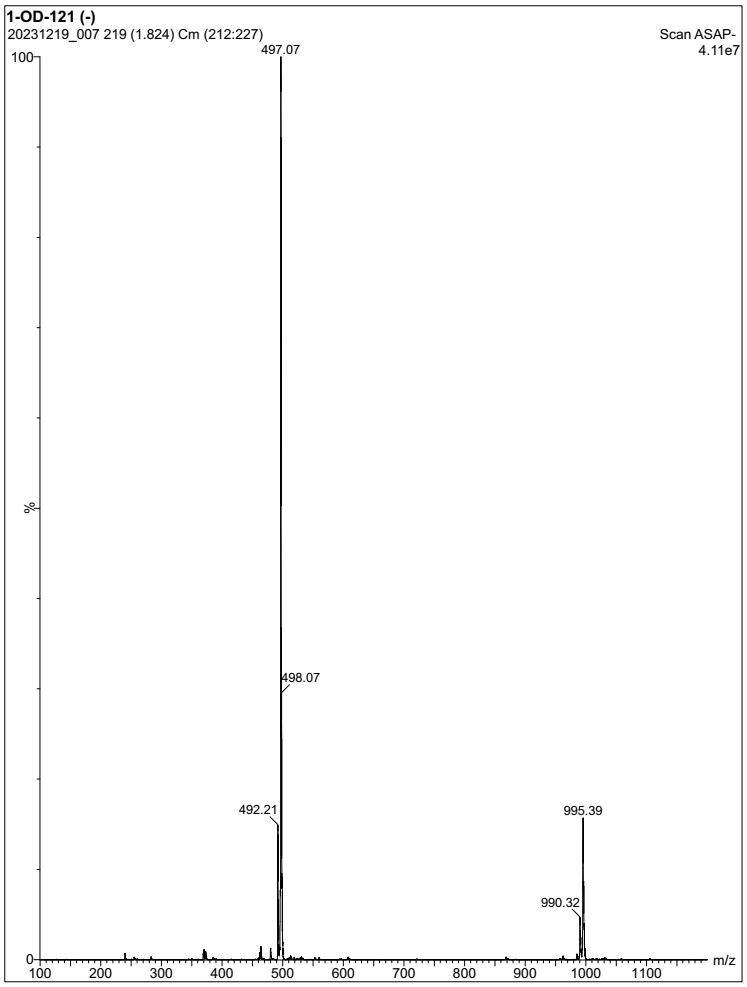

Mass Spectrum of compound **7c** (Negative Mode)


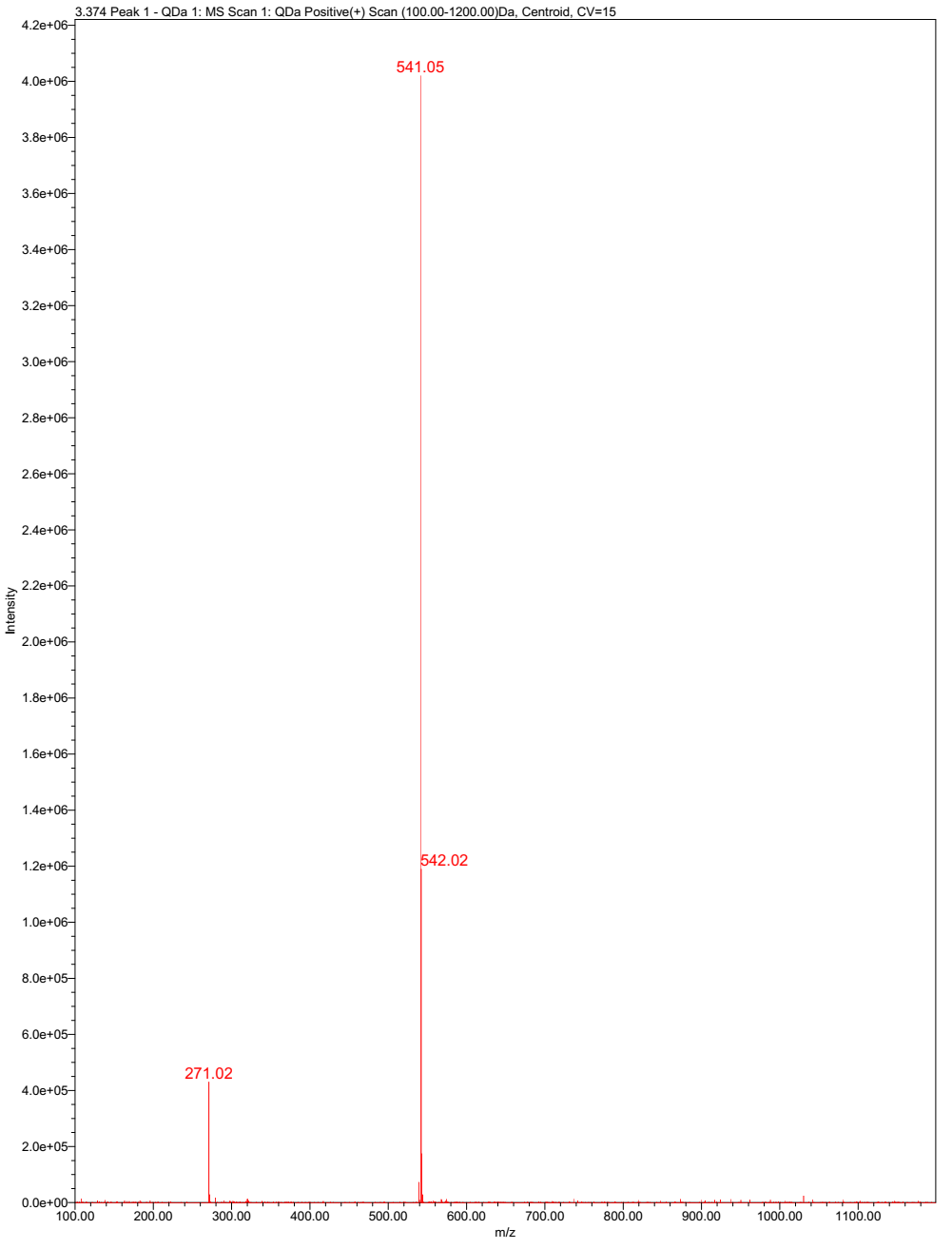

Mass Spectrum of compound **7d** (Positive Mode)

**FTIR Spectra**


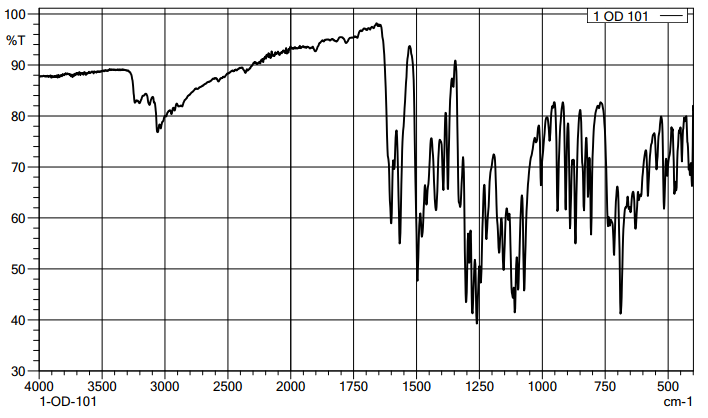

FTIR Spectrum of compound **6**


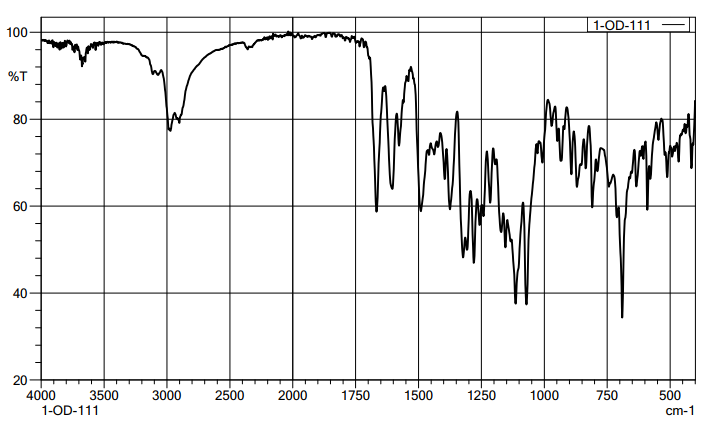

FTIR Spectrum of compound **7a**


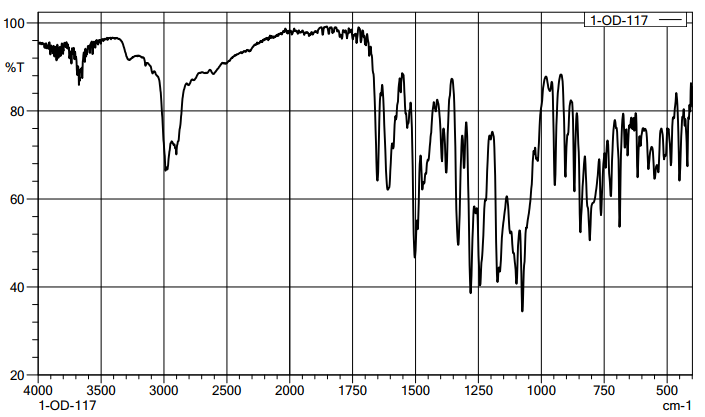

FTIR Spectrum of compound **7b**


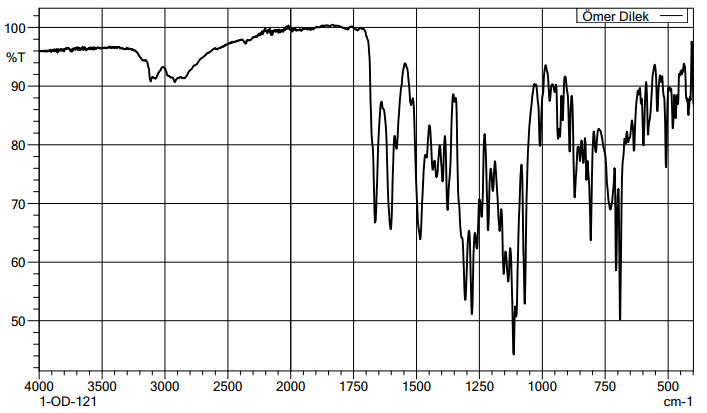

FTIR Spectrum of compound **7c**


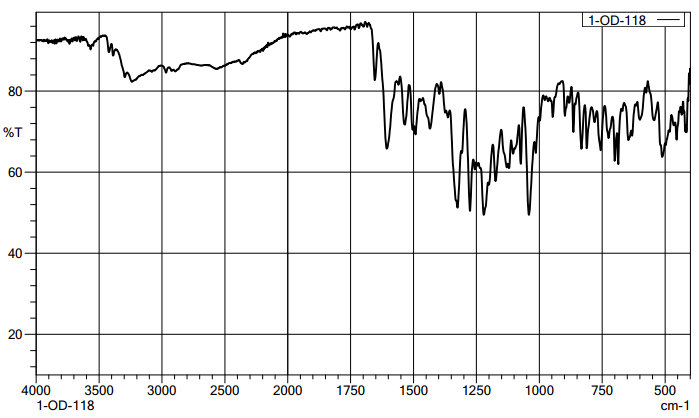

FTIR Spectrum of compound **7d**
